# Supplementary material for: Single-cell analysis of human fetal epicardium reveals its cellular composition and identifies CRIP1 as a modulator of EMT
Source: Stem Cell Reports. 2023 Jun 29;18(7):1421–35. doi: 10.1016/j.stemcr.2023.06.002 (PMC10362506; doi:10.1016/j.stemcr.2023.06.002)
Supplement: Document S1. Figures S1–S5 and supplemental experimental procedures [file mmc1.pdf]

Stem Cell Reports, Volume *18*

Supplemental Information

Single-cell analysis of human fetal epicardium reveals its cellular composition and identifies CRIP1 as a modulator of EMT

Thomas J. Streef, Esmee J. Groeneveld, Tessa van Herwaarden, Jesper Hjortnaes, Marie José Goumans, and Anke M. Smits

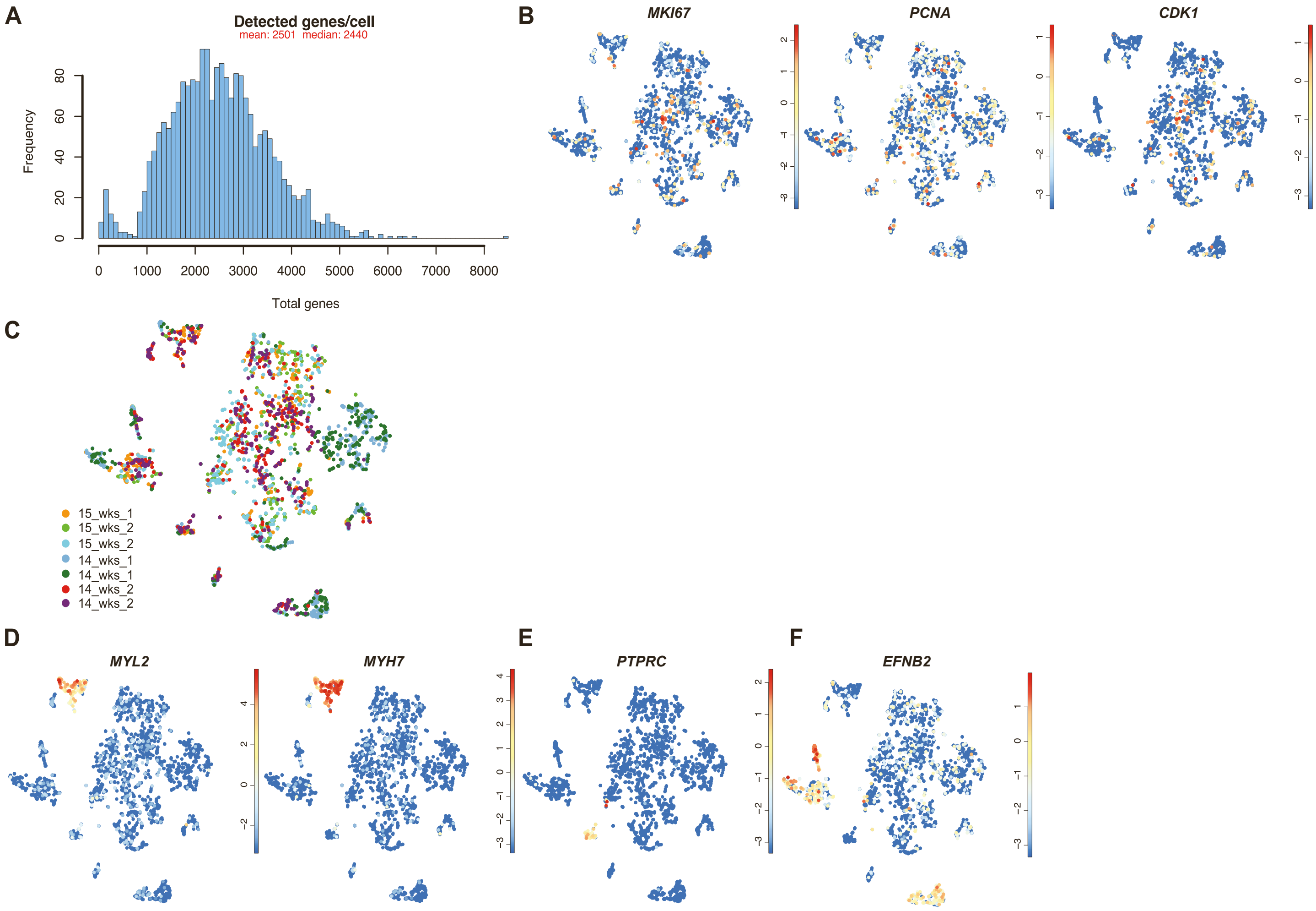

Figure S1. Analysis of clusters in scRNA seq data of isolated cells. Related to Figure 1
(A) Detected genes per cell over all samples. (B) tSNE map of expression of cell-cycle related genes. (C) Distribution of samples across clusters. (D) Expression of markers associated with mature cardiomyocytes. (E) Expression of CD45 (*PTPRC*). (F) Expression of *EFNB2*.

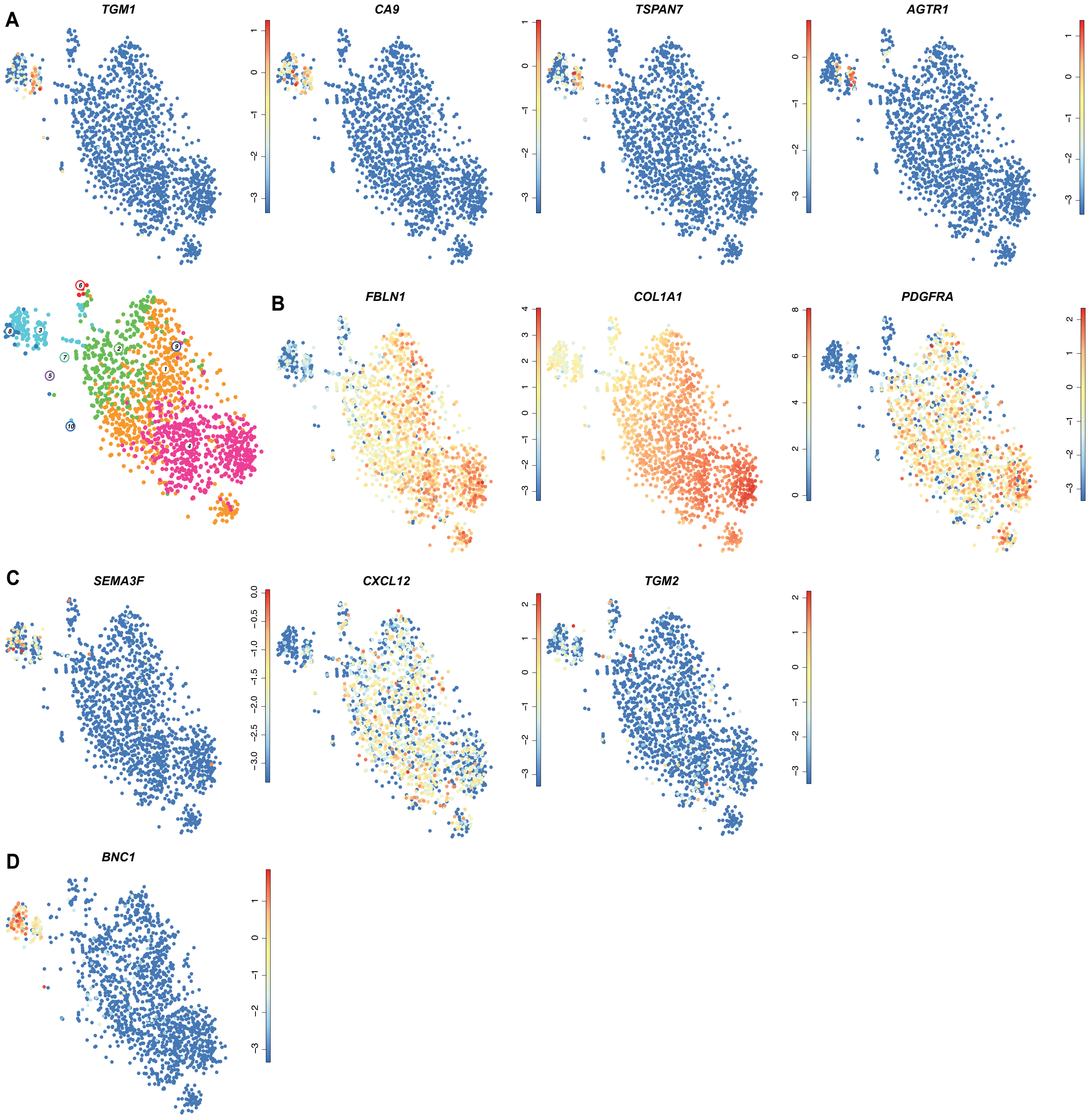

Figure S2. Expression of genes found in subclusters of EACs. Related to Figure 2.
(A) Expression of of markers found in EAC3: *TGM1, CA9, TSPAN7* and *AGTR1*. tSNE map of clustering serves as reference. (B) Expression of ECM-related genes and *PDGFRa* in EACs. (C) Expression of markers for subpopulations found in zebrafish *CXCL12*, *SEMA3F* and *TGM2*. (D) Expression of *BNC1*.

A

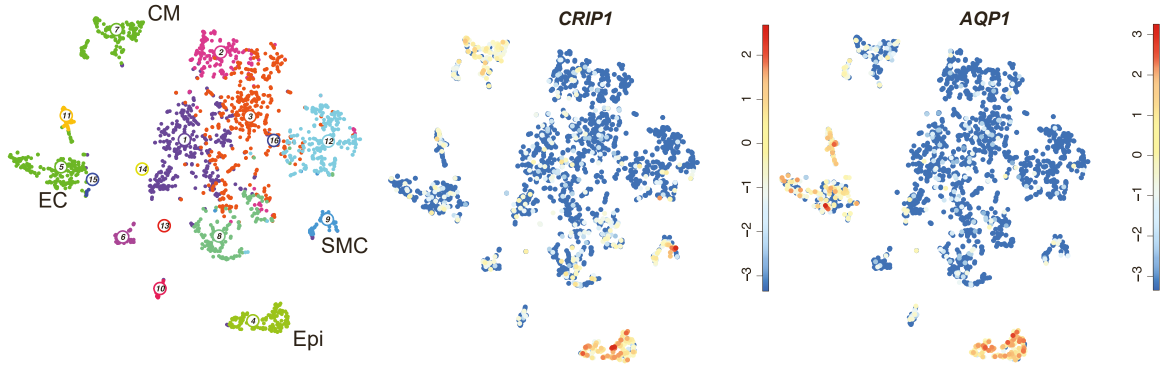

B

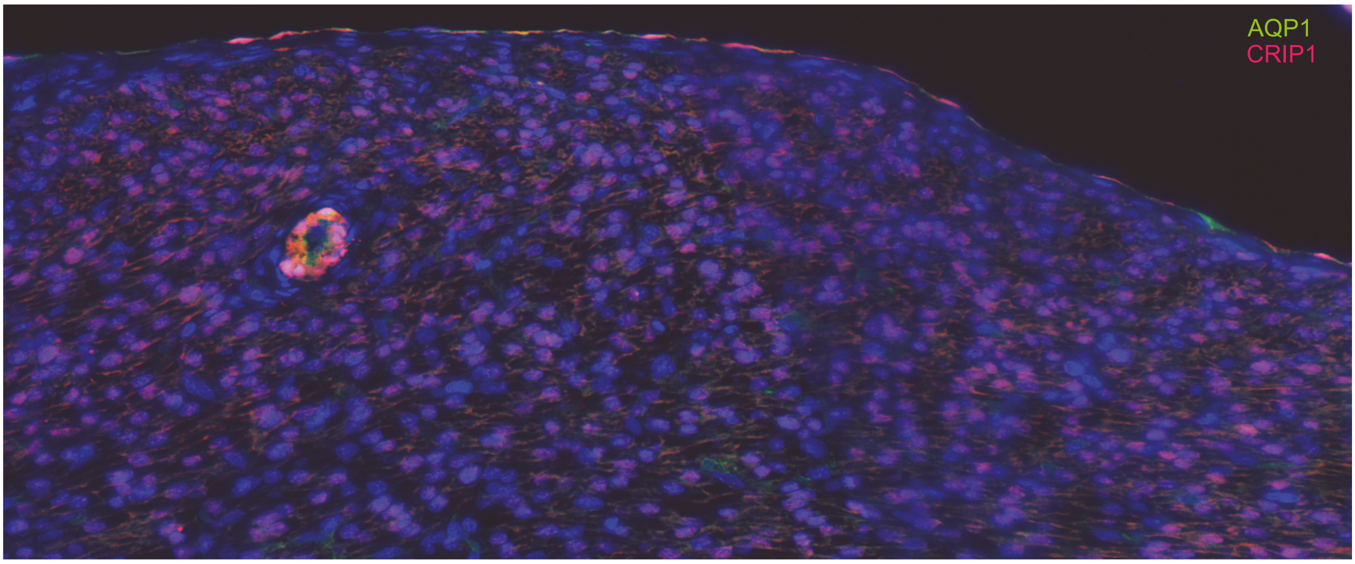

Figure S3. Expression on mRNA and protein level of AQP1 and CRIP1 in human fetal heart. Related to Fig 3.
(A) tSNE map of all clusters and expression of *CRIP1* and *AQP1*. tSNE map of all clusters serves as reference.
(B) Overview of immunohistochemistry of a section of human fetal heart tissue (13 weeks). Counterstain is DAPI.

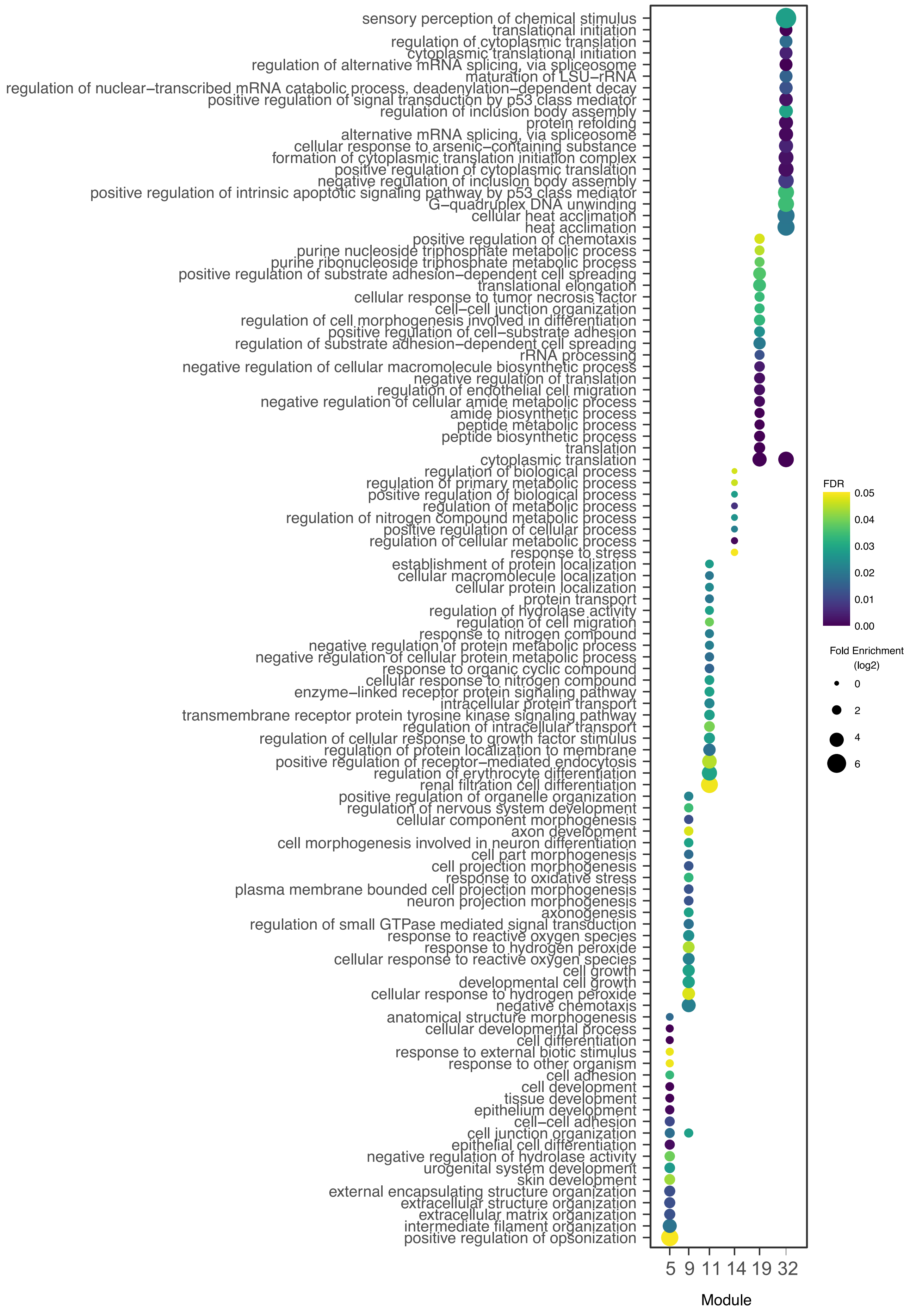

Figure S4. Gene ontology enrichment analysis of genes that have similar expression profile in the pseudotime trajectory. Related to Fig 4. GO term enrichment of genes found in gene expression modules with high expression in epithelial cells and low expression in mesenchymal cells. Color: false discovery rate (FDR), size: enrichment ratio (log2). Modules 1, 2, 3, 6, 7, 10, 12, 13, 15, 16 did not yield any significant GO term enrichment. Modules 4 and 8 only yielded 1 and 5 terms, respectively (data not shown). Top 20 terms with highest fold enrichment are displayed with FDR < 0.05.

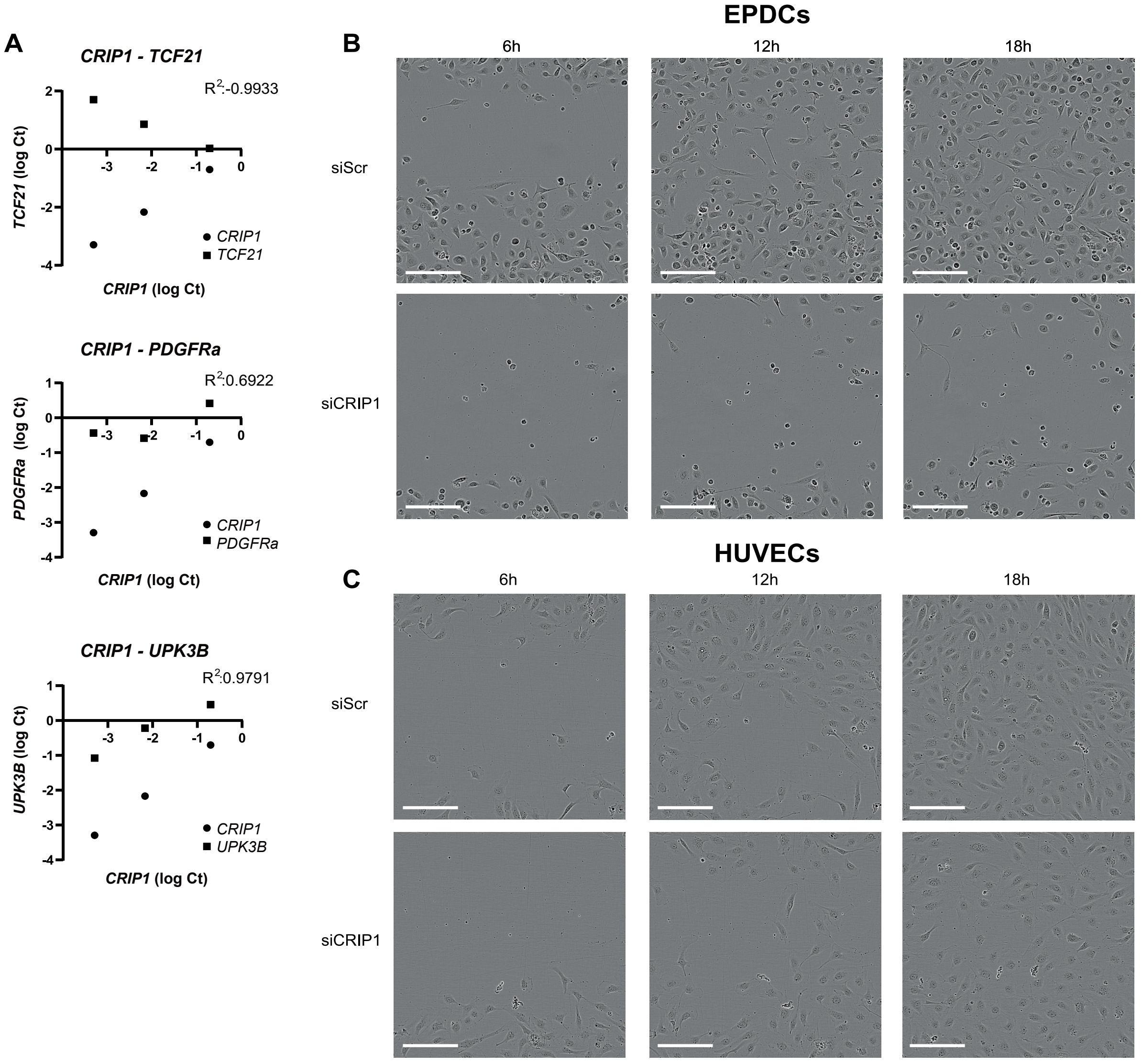

Figure S5. In vitro analysis of the effect of CRIP1 knockdown in EPDCs and HUVECs. Related to Fig 6.
(A)Correlation between *CRIP1* expression and *TCF21, PDGFRa, and UPK3B* in siCRIP1 cells.
(B) Phase contrast images of primary epicardial cells in wound healing assay 6h, 12h and 18h after scratch. Scale bars : 200 μm
(C) Phase contrast images of HUVECs in wound healing assay after 6h, 12h and 18h. Scale bars : 200 μm.

Supplemental experimental procedures

Collection of human cardiac tissue

Human adult heart auricles were collected anonymously as surgical waste from patients undergoing cardiac surgery under general informed consent. Human fetal cardiac tissue was anonymously collected with informed consent from elective abortion material of fetuses with a gestational age between 8 and 20 weeks. This research was carried out according to the official guidelines of the Leiden University Medical Center and approved by the local Medical Ethics Committee (number P08.087). This research conforms to the Declaration of Helsinki.

Isolation of human epicardial cells

Fetal epicardial layers were isolated by separating the epicardium from the underlying myocardium of 4 human hearts aged 14-15 weeks. To create a single cell suspension, the isolated tissue was processed into small pieces and digested during three rounds of Trypsin 0.25%/EDTA incubation (1:1; Serva and USH products) for a total of 30 minutes at 37 °C. The suspension was subsequently passed through a series of syringes of decreasing size (19G to 21G). The cell suspension was passed through a 70-µm cell strainer (BD Falcon), collected and stained for 20 minutes using calcein AM (65-0853, ThermoFisher,) at 100 nM in PBS at RT and subsequently kept on ice until sorting in 384-wells plates.

Sorting and single-cell RNA library prep

Single-cell RNA library prep and plate preparation was performed by Single Cell Discoveries. In short, using a Mosquito® HTS (TTP Labtech) 384 wells plates were filled with 50 nL lysis buffer containing CELseq2-primers, spike-ins and dinucleotide triphosphates and overlaid with mineral oil to prevent evaporation. Plates were stored at -80°C until sequencing. Viable cells were sorted one cell per well into these 384 wells plates using BD FACSAria III, fixed by lysis to preserve expression status, and immediately frozen at -80°C until further processing. cDNA was constructed using the SORT-seq protocol (Muraro et al., 2016).

Analysis of single-cell transcriptome data

RaceID3 was used according to authors instruction (Herman and Grün, 2018). Based on the distribution of the log10 total reads plotted against the frequency, we maintained a cutoff at minimally 1500 reads per cell before further analysis. Moreover, we discarded genes that did not express 3 transcripts in at least 3 cells. We initialized CGenes with *CDK1*, *PCNA*, *MKI67* and ccor = 0.4 to remove cell-cycle associated variability. RaceID3's internal batch correction was applied. To detect outliers, 'findoutliers' was initialized with probthr = 10^{-9}, outlg = 5, outminc = 3. For analysis of subset of epicardial cells, same parameters were applied. 'findoutliers' was run with same parameters, only probthr was set as 10^{-7}.

Gene ontology

Over-representation analysis of differentially expressed genes (fold change > 1.5) for each cluster (or all genes in cases of expression modules) were performed using the non-redundant Biological Process option of Gene Ontology (Ashburner et al., 2000; Carbon et al., 2021). Visualization was done using ggplot2.

Inference of differentiation trajectories

The StemID2 algorithm was used as published previously (Herman and Grün, 2018). In short, StemID2 is an algorithm for identification of differentiation trajectories by inferring links between cell clusters which are more populated by cells with intermediate single-cell transcriptomes than expected by chance. StemID calculates all pairwise cell-to-cell distances (1 – Pearson correlation) and uses this to cluster similar cells into clusters that correspond to the cell types present in the tissue. The StemID algorithm then calculates the number of links between clusters. StemID2 was initialized with cthr = 5, knn = 3 in *nmode.* To define stem cell populations, stem cell score was calculated by StemID2: stem cell score was calculated by multiplying the number of links with the delta-entropy ((number of links * entropy)- minimum median entropy across all clusters)).

Inference of co-expressed gene modules

To identify modules of co-expressed genes along a specific differentiation trajectory, FateID was used (Herman and Grün, 2018). In short, to identify co-expression of genes along a differentiation trajectory to a defined target state (as identified by StemID2), the expression levels in the pseudo-temporally

ordered cells are smoothed by local regression after z-score transformation. These pseudo-temporal gene expression profiles are topologically ordered through computation of a one-dimensional self-organizing map with 1000 nodes. Because of the large number of nodes relative to the number of clustered profiles, similar profiles are assigned to the same node. Only nodes with more than five assigned profiles are retained for visualization of co-expressed gene modules. Neighboring nodes with average profiles with a Pearson's correlation coefficient > 0.85 are merged with common gene expression modules. All functions were initialized with default values.

Inference of a gene regulatory network
GENIE3 computes the regulatory network for each gene independently. It calculates a random forest using genes as predictors. Then, it uses the random forests importance measure as gene association score to indicate a putative regulatory link. The weight of an interaction comes from the importance of an input gene in the predictor for a target gene's expression pattern. Aggregating these weighted interactions over all the genes yields the regulatory network. Genes with a median expression of 0 across all clusters were excluded.

Epicardial cell culture
Epicardial cells for cell culture were isolated as described above and cultured in a mixture of 1:1 Dulbecco's modified Eagle's medium (DMEM-glucose low; Invitrogen) and Medium 199 (M199; Invitrogen) supplemented with 10% heat-inactivated fetal calf serum (FCS; Gibco) and 100 U/ml penicillin/ streptomycin (Gibco). To maintain cells in an epithelial state, the ALK5-kinase inhibitor SB431542 (SB, 5–10 µM; Tocris Bioscience) was added. In experiments aimed at inducing EMT, cells were stimulated with 1 ng/ml transforming growth factor beta 3 (TGFβ3), without SB, for 5 days. TGFβ3 was kindly provided by Prof. P. ten Dijke.

HUVEC cell culture
Human umbilical vein endothelial cells (HUVECs) were cultured in EGM2 (CC-3162, Lonza) supplemented with 2% FCS and 100 U/ml penicillin/streptomycin until confluent and subsequently passaged.

Immunofluorescence staining of human heart tissue
Immunohistochemical staining was performed on formalin-fixed paraffin embedded human fetal and adult atrial cardiac tissue. Tissue was fixed overnight in 4% PFA and then processed for paraffin embedding and sectioned. 6 µm sections were deparaffinized and rehydrated by ethanol gradient and heat-induced antigen retrieval was performed in a pressure cooker for 20 minutes. Sections were washed in PBS/0.1% Tween20 and blocked with block buffer (PBS/2%BSA/0.1% Tween20) for 30 minutes. Sections were incubated with primary antibodies overnight at 4°C diluted in block buffer. Antibodies: WT1 (ab89901, Abcam), AQP1 (sc-25287, Santa Cruz), CRIP1 (PA5-24643, ThermoFisher), TCF21 (HPA013189, Sigma Aldrich), NRK (PA-53566, ThermoFisher), THBS4 (AF2390, R&D Systems), SULF1 (ab32763, Abcam). TCF21 signal was amplified using TSA (NEL700A001KT, PerkinElmer). Secondary antibodies were Donkey Anti-Mouse 555 (A-31570, Life Technologies) and Donkey Anti-Rabbit 647 (A-31572, Life Technologies). Nuclei were stained using DAPI (62248, ThermoFisher Scientific) and slides were mounted using Prolong Gold Antifade (P3630, Invitrogen)

Isolation of mRNA and qPCR
mRNA was isolated using ReliaPrep™ RNA Miniprep Systems (Promega). The mRNA concentration and purity were measured using NanoDrop 1000 Spectrophotometer (Thermo Fisher Scientific) followed by cDNA synthesis using the RevertAid H Minus First Strand cDNA Synthesis Kit (Thermo Fisher Scientific). qPCR was performed in a 384 wells format using SYBR Green (Promega) and run on a CFX384 Touch™ Real-Time PCR Detection System (Bio-Rad). Expression levels were normalized for two reference genes (*HPRT1* and *TBP*) which were designed and tested for robust expression in adult EPDCs and in epithelial and mesenchymal samples using geNorm (Vandesompele et al., 2002). Primers used are displayed below.

Target	Forward (5' - 3')	Reverse (5' - 3')
WT1	CAGCTTGAATGCATGACCTG	TATTCTGTATTGGGCTCCGC
TCF21	CAAGGCCTTCTCCAGACTCA	TCAGGTCACTCTCGGGTTTC
PDGFRa	GAGTGACCATCCTGCTGTGG	GTCTGCGAGCTGTGTCTGTT

POSTN	GACTCAAGATGATTCCCTTTTTACC	GCAACATTCATATAACACAGTCGTTT
SNAI1	CCAGTGCCTCGACCACTATG	CTGCTGGAAGGTAAACTCTGGA
SNAI2	CGGACCCACACATTACCTTGT	TTCTCCCCCGTGTGAGTTCTA
TAGLN	TTCAAGCAGATGGAGCAGGT	TTCAAGCAGATGGAGCAGGT
SMA	CCGGGAGAAAATGACTCAAA	GAAGGAATAGCCACGCTCAG
CRIP1	TCATGCCCAAGTGTCCCAAG	GGTTTGCCTTCGTGCTCAG
BNC1	CCACCGTCAGTGTGACCAAT	CAATCTCCACCTGGCTTGTT
TBP	TGGAAAAGTTGTATTAACAGGTGCT	GCAAGGGTACATGAGAGCCA
HPRT1	CTCATGGACTGATTATGGACAGGAC	GCAGGTCAGCAAAGAACTTATAGCC

siRNA experiments
Prior to adding siRNAs, SB431542 was removed to prevent interference in EMT. ON-TARGETplus SMARTPool siRNAs for CRIP1 (L-016212-00-0005, Dharmacon) or scrambled control (siScr, D-001810-10-05, Dharmacon) were transfected at 25 nM according to manufacturer's instruction without penicillin/streptomycin. After 24h incubation, medium was replaced by standard culture medium supplemented with FCS/penicillin/streptomycin and cells were stimulated for EMT experiments or replated at desired densities for migration experiments.

Migration experiments
Cells were seeded at 20.000 cells per well in a 96-wells plate (Corning) until confluent. Next, WoundMaker^{TM} tool (Essen BioScience) was used to create reproducible scratches in all wells according to manufacturer's instructions. Cells were incubated for 24h in IncuCyte ZOOM^{TM} and photos were taken every two hours. Analysis was performed using IncuCyte Scratch Wound Analysis software module. Statistical analysis for migration in HUVECs was performed with a paired student's t-test.

MTT
Proliferation after siRNA treatment was measured using MTT assay. After siRNA treatment for 24h, 7500 cells were seeded in a 96-wells plate for 24h. 3h prior to analysis, medium was refreshed with EPDC medium with 10% MTT (M5655, Sigma-Aldrich) solution. DMSO was added at the end of the 24h and absorbance was measured in Victor X3 (PerkinElmer) at 595 nm.

Tube formation
Prior to seeding HUVECs, 50 µl of Matrigel (Growth Factor Reduced, 354230, Corning) was incubated in 96-wells plates for 30 minutes. Cells were trypsinized after 24h of incubation with siRNAs (see siRNA interference), and 10.000 cells per well were seeded onto polymerized matrix in normal culture medium. Cells were incubated for 24h in IncuCyte ZOOM^{TM} and photos were taken every two hours. Tube formation was stable at 24h, and images from this timepoint were analyzed with Image J 2.3.0 using Angiogenesis Analyzer (Carpentier et al., 2020).

References

Ashburner, M., Ball, C.A., Blake, J.A., Botstein, D., Butler, H., Cherry, J.M., Davis, A.P., Dolinski, K., Dwight, S.S., Eppig, J.T., et al. (2000). Gene Ontology: tool for the unification of biology. Nat. Genet. *25*, 25–29. https://doi.org/10.1038/75556

Carbon, S., Douglass, E., Good, B.M., Unni, D.R., Harris, N.L., Mungall, C.J., Basu, S., Chisholm, R.L., Dodson, R.J., Hartline, E., et al. (2021). The Gene Ontology resource: enriching a GOld mine. Nucleic Acids Res. *49*, D325–D334. https://doi.org/10.1093/NAR/GKAA1113.

Carpentier, G., Berndt, S., Ferratge, S., Rasband, W., Cuendet, M., Uzan, G., and Albanese, P. (2020). Angiogenesis Analyzer for ImageJ — A comparative morphometric analysis of "Endothelial Tube Formation Assay" and "Fibrin Bead Assay." Sci. Rep. *10*, 1–13. https://doi.org/10.1038/s41598-020-67289-8.

Herman, J.S., Sagar, and Grün, D. (2018). FateID infers cell fate bias in multipotent progenitors from single-cell RNA-seq data. Nat. Methods *15*, 379–386. https://doi.org/10.1038/nmeth.4662.

Muraro, M.J., Dharmadhikari, G., Grün, D., Groen, N., Dielen, T., Jansen, E., van Gurp, L., Engelse, M.A., Carlotti, F., de Koning, E.J.P., et al. (2016). A Single-Cell Transcriptome Atlas of the Human Pancreas. Cell Syst. *3*, 385-394.e3. https://doi.org/10.1016/J.CELS.2016.09.002.

Vandesompele, J., De Preter, K., Pattyn, F., Poppe, B., Van Roy, N., De Paepe, A., and Speleman, F. (2002). Accurate normalization of real-time quantitative RT-PCR data by geometric averaging of multiple internal control genes. Genome Biol. *3*, 1–12. https://doi.org/10.1186/GB-2002-3-7-RESEARCH0034/COMMENTS.
