# Supplementary material for: Single-cell analysis of human fetal epicardium reveals its cellular composition and identifies CRIP1 as a modulator of EMT
Source: Stem Cell Reports. 2023 Jun 29;18(7):1421–35. doi: 10.1016/j.stemcr.2023.06.002 (PMC10362506; doi:10.1016/j.stemcr.2023.06.002)
Supplement: Document S2. Article plus supplemental information [file mmc7.pdf]

Stem Cell Reports
Article
OPEN ACCESS

Single-cell analysis of human fetal epicardium reveals its cellular composition and identifies CRIP1 as a modulator of EMT

Thomas J. Streef,[1] Esmee J. Groeneveld,[1] Tessa van Herwaarden,[1] Jesper Hjortnaes,[2] Marie José Goumans,[1] and Anke M. Smits[1,*]

[1]Department of Cell and Chemical Biology, Leiden University Medical Center, Leiden, the Netherlands
[2]Department of Cardiothoracic Surgery, Leiden University Medical Center, Leiden, the Netherlands
*Correspondence: a.m.smits@lumc.nl
https://doi.org/10.1016/j.stemcr.2023.06.002

SUMMARY

The epicardium plays an essential role in cardiogenesis by providing cardiac cell types and paracrine cues to the developing myocardium. The human adult epicardium is quiescent, but recapitulation of developmental features may contribute to adult cardiac repair. The cell fate of epicardial cells is proposed to be determined by the developmental persistence of specific subpopulations. Reports on this epicardial heterogeneity have been inconsistent, and data regarding the human developing epicardium are scarce. Here we specifically isolated human fetal epicardium and used single-cell RNA sequencing to define its composition and to identify regulators of developmental processes. Few specific subpopulations were observed, but a clear distinction between epithelial and mesenchymal cells was present, resulting in novel population-specific markers. Additionally, we identified CRIP1 as a previously unknown regulator involved in epicardial epithelial-to-mesenchymal transition. Overall, our human fetal epicardial cell-enriched dataset provides an excellent platform to study the developing epicardium in great detail.

INTRODUCTION

The heart is covered by a mesothelial cell layer known as the epicardium. This single-cell, thick layer derives from the proepicardium, a transient population of progenitor cells at the venous pole of the developing heart (Niderla-Bielinska et al., 2019). Once the epicardium is established, a subset of epicardial cells undergoes epithelial-to-mesenchymal transition (EMT) and delaminates from the epicardial layer to populate the subepicardial space and to invade the myocardium. These epicardial-derived cells (EPDCs) have been reported to differentiate into various cell types, including fibroblasts, pericytes (PCs), and smooth muscle cells (SMCs) (Acharya et al., 2012; Cai et al., 2008; Gittenberger-de Groot et al., 1998; Grieskamp et al., 2011). While cellular contributions of EPDCs to endothelial cell (EC) (Carmona et al., 2020) and cardiomyocyte (CM) (Zhou et al., 2008) populations have been reported, an epicardial contribution to these cell types is likely limited (Tian et al., 2015). Additionally, the epicardium and EPDCs produce paracrine cues such as fibroblast growth factors and retinoic acid, thereby orchestrating developmental processes including proliferation and maturation of CMs and ECs (Masters and Riley, 2014). The crucial role of the epicardium in cardiac development is underlined by experiments where *in vivo* inhibition of epicardial outgrowth led to severely impaired myocardial and coronary vessel development (Gittenberger-de Groot et al., 2000). In the adult heart, the epicardium is a quiescent layer, but it is reactivated after ischemic injury (Zhou et al., 2011) with epicardial cells partly recapitulating developmental capacities such as expression of developmental genes, proliferation, and EMT (forming the subepicardium) but with little migration into the tissue. Although the adult epicardium after ischemic injury displays limited contribution to cardiac tissue formation, its activation is essential to the post-injury response (Duan et al., 2011; Smart et al., 2011). Since the adult epicardium partly recapitulates the properties of its embryonic counterpart, increasing our understanding of the epicardium and its composition during development may reveal novel approaches to improve the contribution of epicardial cells in the post-injury response.

The epicardium has been characterized by several transcription factors such as Wilms' Tumor 1 (WT1), T-box 18 (TBX18), and transcription factor 21 (TCF21) (Cai et al., 2008; Kikuchi et al., 2011; Moore et al., 1998). In mice and chick embryos, these markers are not uniformly expressed in the epicardium or the subepicardial mesenchyme, implying a degree of cellular heterogeneity (Braitsch et al., 2012). Recently, single-cell RNA sequencing (scRNA-seq) has provided key insights into *in situ* epicardial heterogeneity and function in organisms such as mouse and zebrafish (Lupu et al., 2020; Weinberger et al., 2020). In mouse embryos, there is a considerable overlap of epicardial markers, and cell fate of EPDCs does not depend on the expression of the epicardial transcription factors WT1, TBX18, or TCF21 (Lupu et al., 2020). In other studies, epicardial heterogeneity was thought to reflect developmental progression and EMT, rather than to indicate the presence of distinct subpopulations (Streef and Smits, 2021). In contrast, functional subpopulations were identified in zebrafish: knockout of genes specific to these

This is an open access article under the CC BY license (http://creativecommons.org/licenses/by/4.0/).

subpopulations affected epicardial cell number, migration, and homing of non-epicardial cells (Weinberger et al., 2020). In humans, we are only starting to unravel the composition of the epicardium and subepicardial mesenchyme. A recent study by Knight-Schrijver et al. compared the composition of the fetal epicardium during several stages of development to the adult heart and found that the mesothelial cell state of the epicardium persisted into adult life, while other cell stages were transient (Knight-Schrijver et al., 2022). These data were mainly collected from whole heart samples, in which the epicardium is only a small proportion of cells. To fully appreciate the composition and the processes that occur within the epicardium, a more detailed analysis is required. Here we used a previously established protocol to separate the human (sub)epicardium from the underlying cardiac tissue (Dronkers et al., 2018) and generated an epicardial cell-enriched dataset to analyze the composition and function of the human fetal epicardium. scRNA-seq revealed limited heterogeneity within the epicardium, but we identified several novel markers for epithelial and mesenchymal stages of epicardial cells. By focusing on the process of EMT, we discovered CRIP1 as a potential regulator of epicardial EMT, demonstrating that our dataset can be used to identify functional modulators of developmental processes.

RESULTS

Single-cell sequencing data enriched for epicardium-associated cells

To specifically study the human fetal epicardium, we manually separated the epicardial layers from the underlying tissue of four human fetal hearts staged 14 and 15 weeks of gestation. Tissues were dissociated into a single-cell suspension, and live calcein$^+$ cells were sorted into 384-well plates (2,137 cells) using fluorescence-activated cell sorting (FACS) for single-cell sequencing (Figure 1A). Spike-ins and mitochondrial reads were discarded, and 2,501 genes were detected per cell on average (Figure S1A). Subsequent data analysis was performed using RaceID3 (Herman and Grün, 2018). After quality control, 2,073 cells remained for further analysis. Cell clusters were visualized by applying unsupervised t-distributed stochastic neighbor embedding (tSNE), revealing 16 clusters in our dataset (Figure 1B). To avoid cell-cycle associated variability, genes correlating to expression of *PCNA*, *MKI67*, and *CDK1* were not included in clustering (Figure S1B). The distribution of samples across clusters was verified (Figure S1C). Clusters >10 cells were identifiable based on the expression of cell-type-specific markers (Figure 1C and Table S1).

Epithelial epicardial cells were identified in cluster 4 based on the expression of *UPK3B, MSLN*, and *KRT19* (Bochmann et al., 2010) and represented 141 cells (6.8% of total cells). The subepicardium is composed of mesenchymal cells that derive from the epicardial epithelium (Moore-Morris et al., 2016). We distinguished mesenchymal cells based on associated genes (*POSTN, DPT, PDGFRA*), and we found that these cells were abundantly present in clusters 1, 2, 3, 8, and 12 (65.3% of isolated cells). In total, 72.1% of the cells within our isolate were related to the (sub)epicardium.

Our isolation method cannot fully prevent co-isolation of cardiac tissue, and indeed, we observed other cardiac cell types within our dataset. CMs were present in cluster 7 (*TNNI3*, *ACTC1*, and *MYL4,* 7.9%). Notably, a subset of CMs had a relatively low expression of *MYH7* and *MYL2* (Figure S1D), indicating that cluster 7 contained mature and immature cardiomyocytes (Guo and Pu, 2020). Cluster 10 (1.7%) consisted of cells expressing *SOX10*, *S100b*, and *NRXN*, suggesting derivation from the neural crest. Cluster 5 held a hematopoietic *CD45*$^+$ population (Figure S1E), which contributes to the epicardium of the developing mouse heart (Balmer et al., 2014). Other cardiac cell types included SMCs/PCs expressing *RGS5* and *ACTA2* (3%, cluster 9). ECs expressing *PECAM* and *VWF* clustered separately from the epicardium(-derived) cells in clusters 5, 11, and 15. These three endothelial clusters were closely related, and subclustering may have occurred due to a higher expression of artery-specific marker *EFNB2* in cluster 11 (Figure S1F) (Xu et al., 2018). Nevertheless, all three clusters appeared to be endothelial and comprised a total of 11.9% of the total population. Several small clusters were identified: cluster 13 cells (six cells) were likely red blood cells based on the expression of *HBG1/2,* and cluster 14 expressed neuronal markers *CHRNA3* and *SYN2* (five cells). Cluster 16, consisting of two cells, was unidentifiable. Overall, our isolation method resulted in a high yield of epicardium-associated cells that were selected for further analysis.

Expression of marker genes in isolated epicardial cells and their derivatives

We explored the composition of the human fetal epicardium in more detail by excluding cardiac cell types (CMs, ECs), CD45$^+$, and neural crest cells (Figure 2A). The remaining enriched subset of epicardium-associated cells (EACs) was used for separate clustering and analysis (Figure 2A). The threshold for clustering was set to discriminate beyond the epithelial-mesenchymal traits. Epithelial cells were identified based on the combined expression of *UPK3B*, *MSLN*, and *KRT19* (Bochmann et al., 2010), revealing that these cells are now present in two clusters: EAC8 and 3. Mesenchymal cells comprised the largest population within our subset and based on combined expression of markers *DPT*, *POSTN*, and *PDGFRa* were shown to be

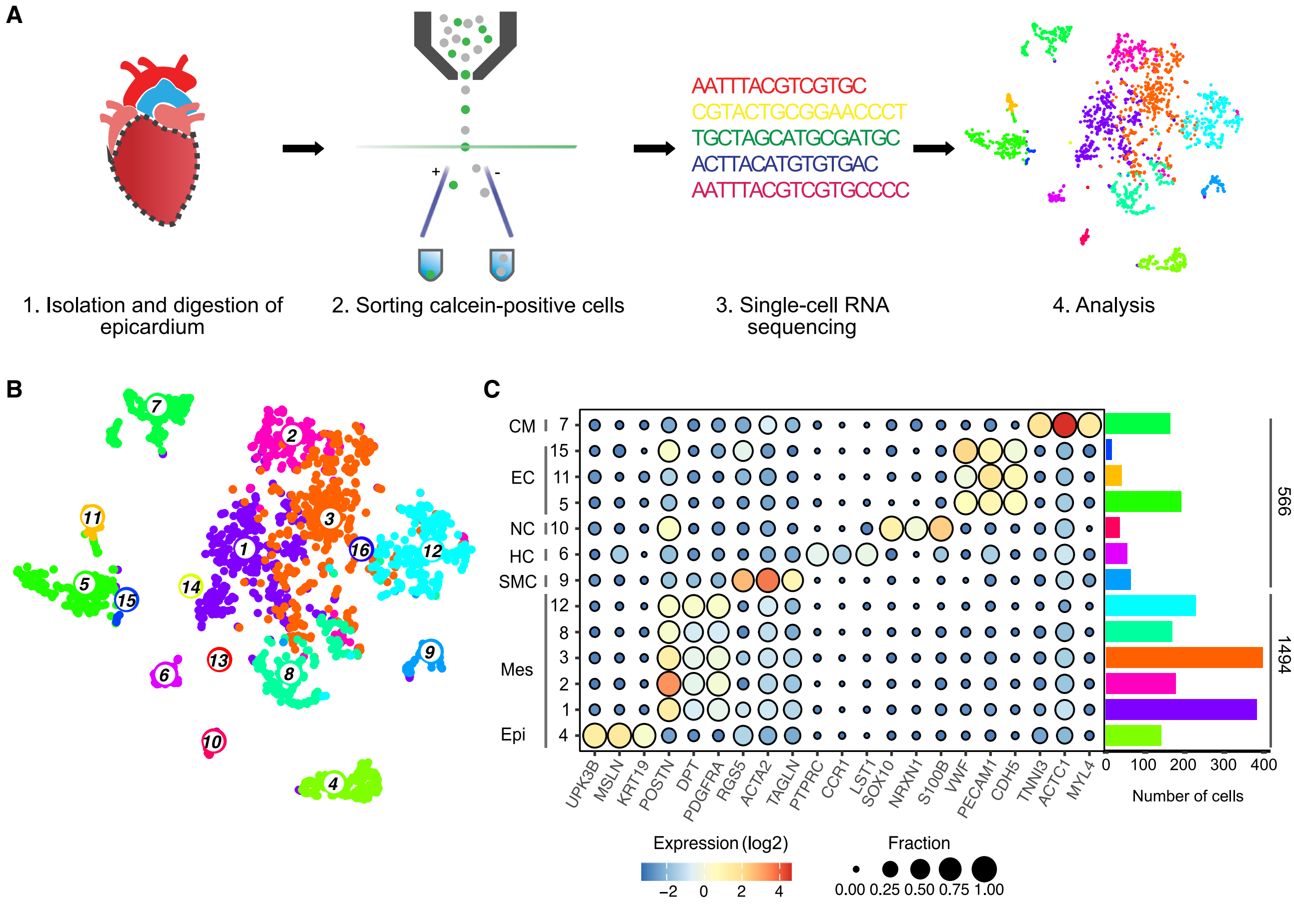

Figure 1. Experimental workflow and tSNE visualization of fetal epicardial and cardiac tissue-related subpopulations
(A) Experimental workflow.
(B) tSNE map shows 16 subpopulations.
(C) Expression dotplot of clusters and their respective markers. Dot size represents fraction of cells expressing the marker, and fill color corresponds to expression levels. Right: a barplot depicts the number of cells in each cluster. Colors correspond to tSNE map in (B). Clusters <10 cells are not shown. Epi, epicardium; Mes, mesenchymal cells; SMC, smooth muscle cells; HC, hematopoietic cells; NC, neural crest; EC, endothelial cells; CM, cardiomyocytes. See also Figure S1.

present in three distinct clusters: EAC1, 2, and 4, which may suggest some degree of heterogeneity. SMCs were retrieved in cluster EAC6 (*RGS5, ACTA2, THBS4*) (Figure 2B).

In line with reports on mouse and chick embryos, *WT1*, *TCF21*, and *TBX18* were non-homogenously expressed in our EACs. Importantly, co-expression of these transcription factors was observed in several cells (Figure 2C). Specifically, *WT1* was primarily found in clusters EAC3 and 8 and at a lower level in some mesenchymal cells, while *TBX18* was expressed in both epithelial and mesenchymal populations. *TCF21* was expressed highest in the mesenchymal populations, with low expression in a small number of cells in cluster EAC8 and low expression in cluster EAC3 (Figure 2C). These observations indicate that for the human epicardium, *WT1*, *TBX18*, and *TCF21* do not distinguish epithelial from mesenchymal cells derived from the epicardium. Furthermore, although expression is heterogeneous in these cells, it is insufficient to cause distinct clustering, and they do not appear to be indicators of epicardial heterogeneity. Since *WT1* and *TBX18* expression decreases in epicardial cells after EMT and *TCF21* increases (Lupu et al., 2020), their expression pattern might be related to the process of EMT.

To assess heterogeneity within the epicardium-related subgroups, we performed differentially expressed gene (DEG) analysis. DEGs were analyzed within the epithelial clusters (EAC3 vs. EAC8; all significant DEGs are listed in Table S2). The expression profiles across the individual clusters of the 10 DEGs with the highest fold change (fc) are displayed (Figure 2D). Interestingly, there were no genes that differentiated EAC3 from EAC8. Although EAC3 had a relatively high expression of genes encoding for ribosomal

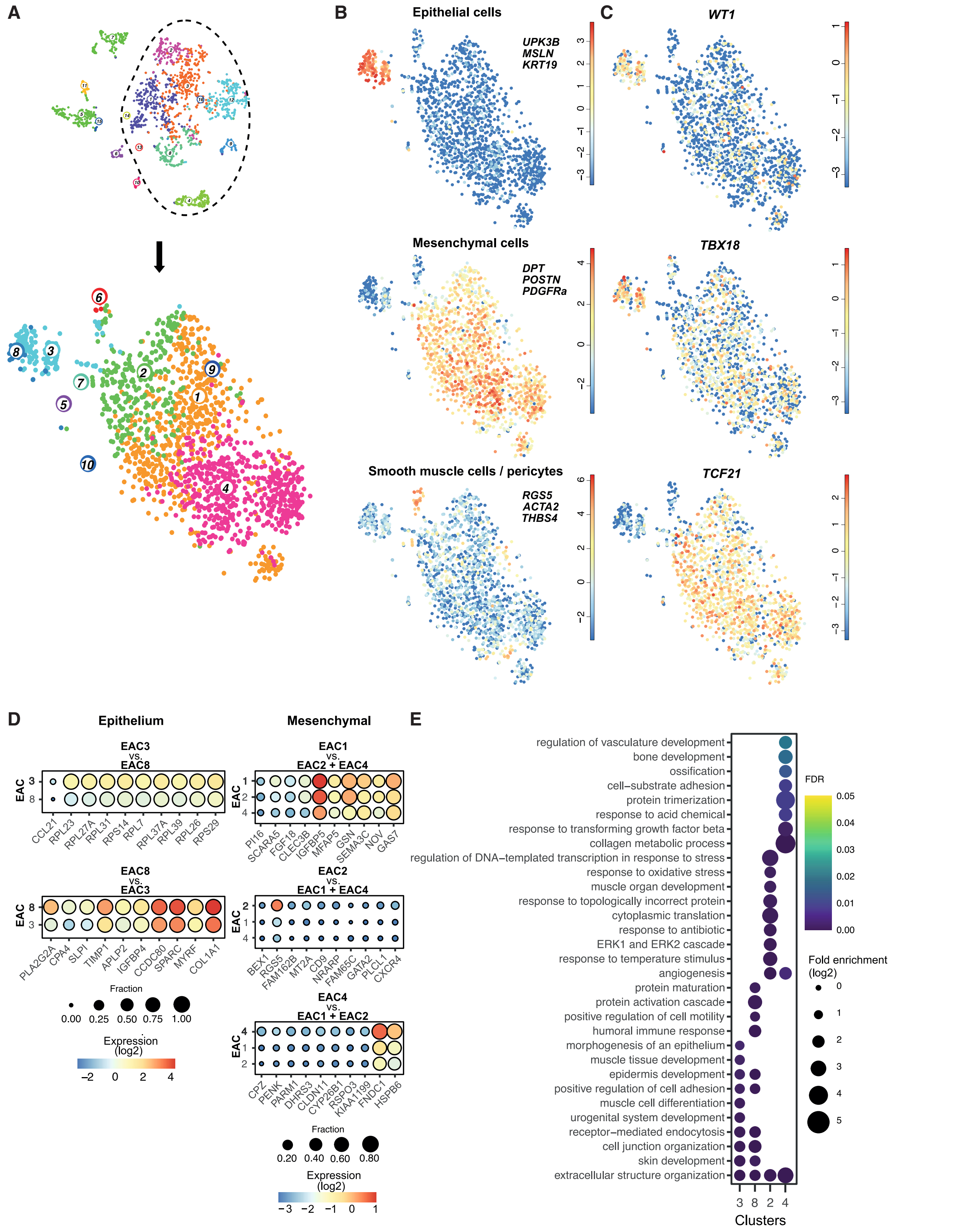

(legend on next page)

proteins (Figure 2D), these were also present in EAC8 (Figure 2D), and the level was comparable to mesenchymal clusters EAC1 and 2 (data not shown). EAC8 displayed a higher expression level of *CPA4* and *PLA2G2A* (Figure 2D), but these genes were not exclusive to this cluster as they are also present in EAC3, making it unlikely that these markers define a distinct subpopulation. Further inspection of the DEGs in EAC3 against all other clusters (Table S1) revealed that *TGM1* was expressed primarily in EAC3, and this cluster was also enriched for *AGTR1, CA9*, and *TSPAN7* (Figure S2A). Within the mesenchymal populations, we compared each cluster to all remaining cells inside its subgroup (e.g., EAC1 versus EAC2+EAC4). All significant DEGs are listed in Table S2. Analyzing the mesenchymal subpopulations revealed that there were few distinguishing markers present (Figure 2D and Table S2). EAC1 had two DEGs with an fc > 2 compared with EAC2+EAC4 (*SCARA5, PI16*, Table S2). EAC2 revealed a higher expression of the SMC marker *RGS5*. EAC4 had a relatively high expression of *FNDC1* and *HSPB6* (Figure 2D) and was also enriched for *PDGFRA,* a driver of fibroblast cell fate (Smith et al., 2011). Additionally, EAC4 had high expression of ECM-encoding genes, such as *COL1A1* and *FBLN1* (Figure S2B). The small number of DEGs between the clusters EAC1, 2, and 4, and the high expression of genes associated with mesenchymal cell function (collagens*, PDGFRa, POSTN*; Figures 2B and S2B), suggested that a functional difference was limited. This was confirmed by Gene Ontology (GO) term analysis on DEGs in all clusters (Figure 2E); EAC3 and EAC8 were enriched for biological processes related to the epithelial layer and the formation of muscle tissue. EAC2 could not be functionally characterized based on GO terms, while EAC1 did not have sufficient DEGs to have any significant GO terms. EAC4 was enriched for genes related to mesenchymal cell functions (collagen metabolic processes, response to TGFβ). Based on these data, EAC4 is the most fibroblast-like population within our dataset.

To investigate if subpopulations observed in other species or models persisted in our data, we focused on expression of markers of epicardial subpopulations that were described in zebrafish (*TGM2*, *SEMA3F*, *CXCL12*) (Weinberger et al., 2020). Overall, these genes were primarily expressed throughout EAC3 and EAC8. Interestingly, while *CXCL12* was proposed to be expressed in the epithelial epicardium in zebrafish embryos, we observed a higher expression of this chemokine in mesenchymal clusters EAC1, 2, and 4 (Figure S2C). This observation correlates with recent studies showing that mesenchymal epicardial cells express *cxcl12a* and mural cells express *cxcl12b* in regenerating adult zebrafish hearts (Xia et al., 2022). Recently, basonuclin (*BNC1*) has been described as regulator of *WT1* and *TCF21* expression in human pluripotent stem cell-derived epithelial epicardial cells (Gambardella et al., 2019). In agreement, we found *BNC1* highly expressed in EAC3 and EAC8 and low expression in mesenchymal cells (Figure S2D).

Identification of markers in the epicardium and its derivatives

While differences within the epithelial and mesenchymal populations were not apparent, large differences between epithelial and mesenchymal cells were observed, allowing the identification of markers with a specific expression profile for these populations. Based on DEG analysis, several genes were highly expressed in epithelial clusters (EAC3 and EAC8) in comparison with mesenchymal clusters EAC1, 2, and 4. This included established genes but also less reported ones such as *SULF1*, *CRIP1*, *AQP1*, and *C3* (Figure 3A and Table S3). Mesenchymal subpopulations displayed a differential expression of several ECM-related genes as well as *C7*, *NRK*, *GPC3*, and *DLK1* (Figure 3A and Table S3). To establish the specificity of these markers, we performed *in situ* validation on the protein level in human fetal heart tissue (Figure 3B). Immunohistochemistry confirmed a distinct epithelial signal of AQP1 on the membrane and expression of SULF1 and CRIP1 in the cytosol of cells in the epicardial layer. Additionally, AQP1 and CRIP1 were observed in ECs and SMC, in agreement with the expression patterns in the total sequencing data of all isolated cells (Figure S3A). To further validate our dataset, we investigated the protein expression of *THBS4* as a marker of the SMC population that demarcated EAC6 (Table S1). As expected, $THBS4^{+}$ cells were observed closely located to vessels (Figure 3B). Immunostaining was also performed for mesenchymal markers TCF21 and NRK, since the latter appeared to represent a novel marker of EPDCs. Both markers were observed primarily within the subepicardial space as is expected for epicardial-derived mesenchymal cells. NRK (Nik-related kinase) has not yet been described as a marker for the mesenchymal population, but its

Figure 2. Analysis of epicardium-associated cells (EACs) reveals limited heterogeneity
(A) Top: dashed line depicts cells that were used for subsequent analysis. Bottom: tSNE map of EACs and SMCs.
(B) Expression of epithelial markers (top), mesenchymal markers (middle), and SMC markers (bottom). Expression scale is log2.
(C) Expression of common markers of the epicardium in EACs. Expression scale is log2.
(D) Expression dotplot of DEGs in epithelial and mesenchymal subclusters. The top rows show the 10 DEGs with highest fc compared with the remaining cells in the subcluster.
(E) GO term enrichment of DEGs in EACs. Color, false discovery rate (FDR); size, enrichment ratio (log2). See also Figure S2.

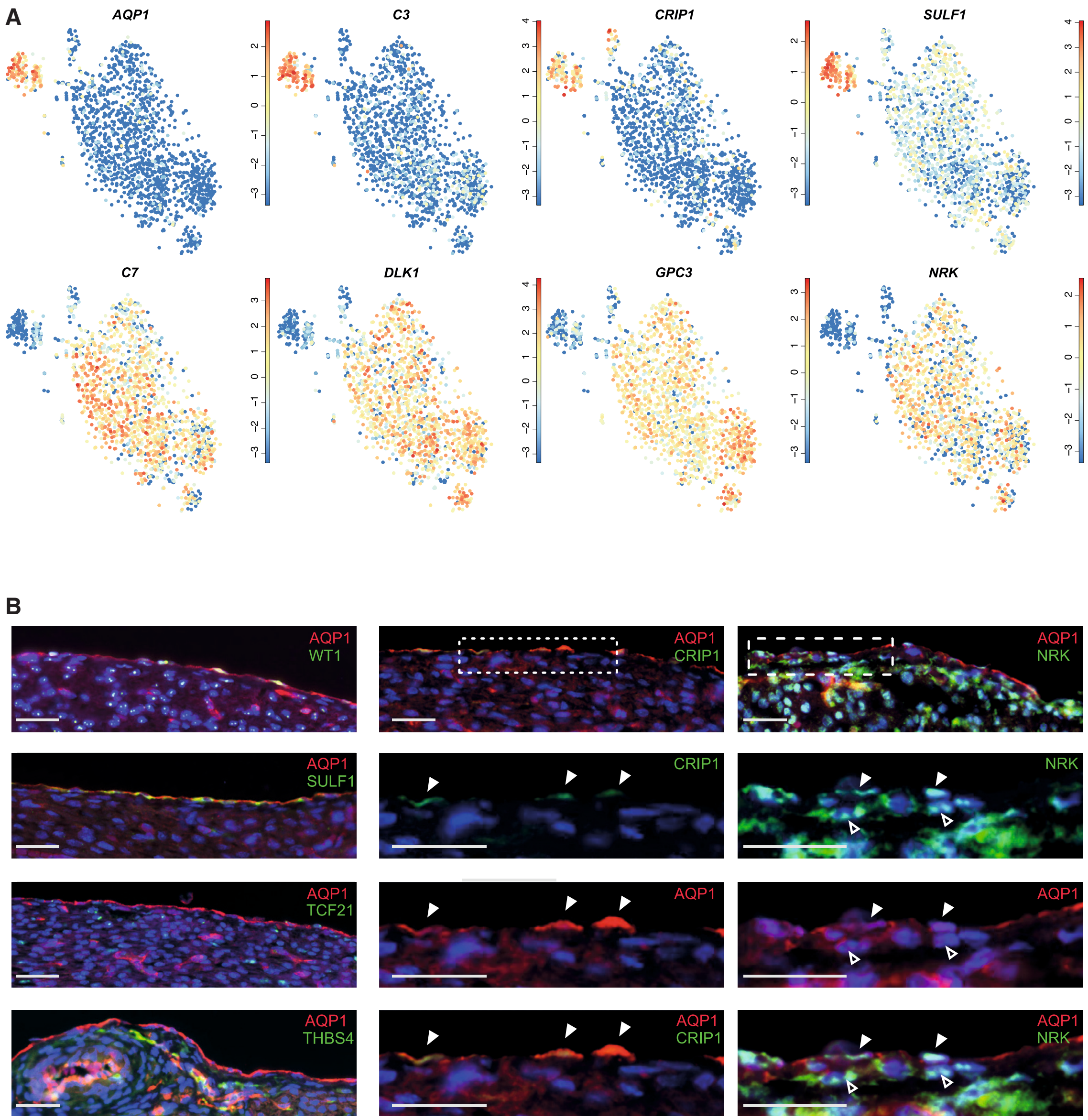

Figure 3. Expression and spatial validation of epithelial and mesenchymal markers

(A) tSNE plots showing expression of (novel) markers in the epithelial (EAC3, 8) and mesenchymal (EAC1, 2, and 4) subpopulations. Expression scale is log2.

(B) Immunohistochemistry with established (WT1, AQP1) or novel markers on human fetal heart tissue. Small dashes: magnification of epicardial section of CRIP1 staining. Large dashes: magnification of epicardial section of NRK staining. Solid arrowheads: epithelial expression. Open arrowheads: mesenchymal expression. Counterstain is DAPI. Scale bar: 25 μm. See also Figure S3.

specificity was confirmed by the combination of the scRNA-seq data and protein expression within the (sub) epicardium. Interestingly, NRK$^+$ cells were also present within the outer epicardium (Figure 3B), suggesting epithelial cells turning on mesenchymal features and retention of these transitioning cells within the epicardium as previously described in developing chicken hearts (Mantri et al., 2021). Overall, we were able to differentiate between epicardial cells with an epithelial and mesenchymal transcriptome, but within these classifications, we found limited heterogeneity based on DEGs and GO analysis.

Differentiation trajectories in epicardial and epicardium-derived cells

We hypothesized that the subpopulations within the epithelial and mesenchymal populations could result

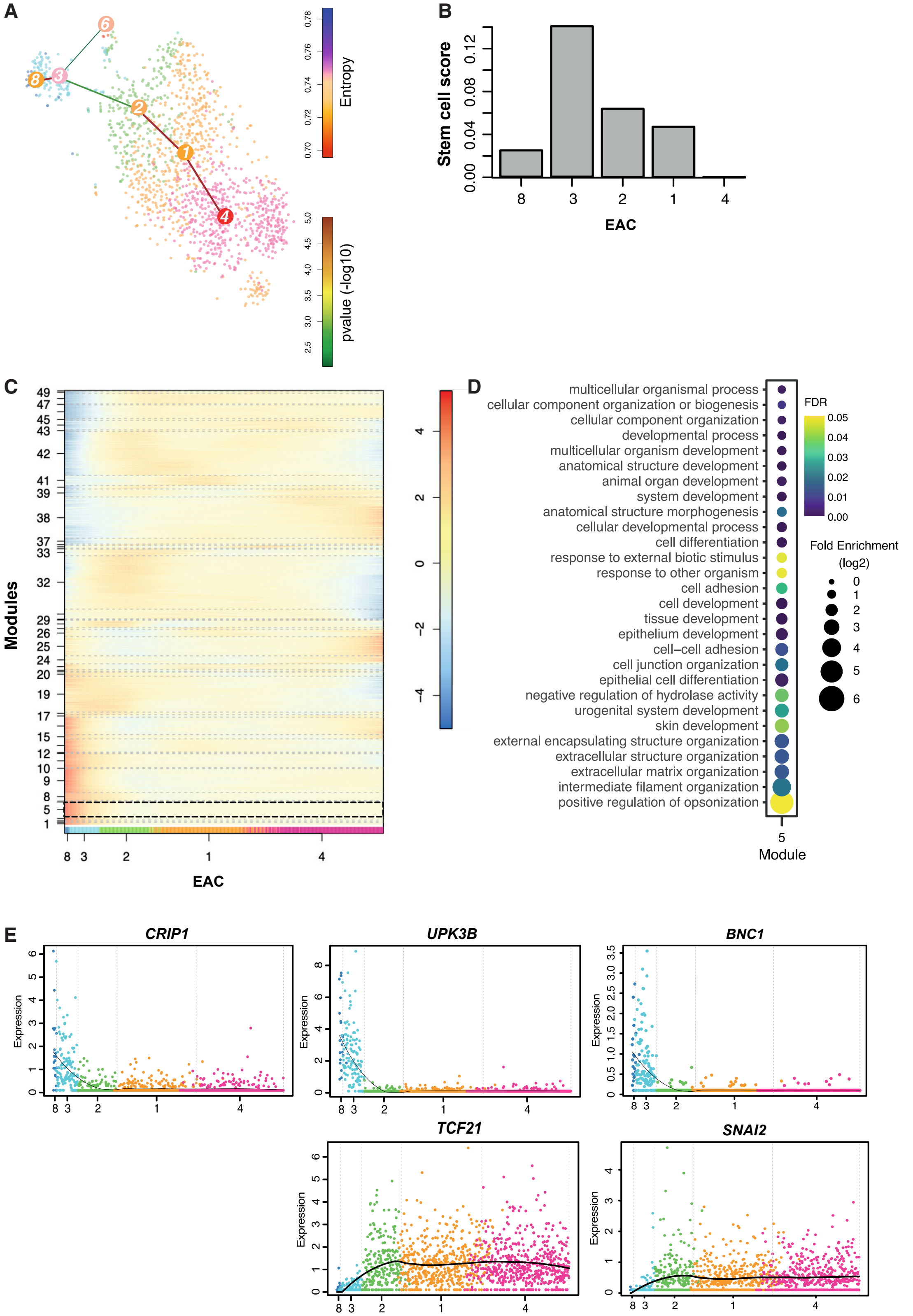

(legend on next page)

from the ongoing process of EMT during cardiac development and the subsequent differentiation into fibroblast and SMCs. To explore these processes, cells were ordered in pseudotime using StemID2 (Herman and Grün, 2018). A potential differentiation path was detected between epithelial and mesenchymal cells; we identified EAC8 as the starting point with EAC4 as the end of the differentiation trajectory (Figure 4A). This path appears to represent epicardial cells undergoing EMT and differentiating into mesenchymal cells with endpoint cluster 4, the most fibroblast-like cells according to our DEG analysis (e.g., *FBLN1, COL1A1, PDGFRA*; Figure S2B). As expected, a differentiation trajectory toward SMCs (EAC3 to EAC6; Figure 4A) was also observed. Next, stem cell scores per cluster were determined, and these indicated that epithelial cluster EAC3 displayed the highest degree of "stemness" (Figure 4B). Based on the combination of differentiation trajectories and the stem cell score, EAC3 should have the highest differentiation potential and could represent a progenitor population. To identify genes with a similar expression profile during differentiation from epithelium to mesenchymal cells, genes were ordered into a self-organizing map (SOM) resulting in 50 modules (Figure 4C). Since EAC3 was suggested to be the most dynamic population, we focused on modules containing those genes that were differentially expressed in EAC3 (Table S1). Genes in module 5 largely overlapped with DEGs (fc > 2) from EAC3 (87 out of 93 genes) (Table S4), including genes related to EMT and differentiation such as *ALDH1A2* and *BNC1* (Gambardella et al., 2019; von Gise et al., 2011). GO analysis of genes in module 5 associated with epithelial development and epithelium differentiation (Figure 4D). Other modules that displayed a high expression in EAC3 and low in EAC4 (modules 9, 11, 14, 19, and 32) were enriched for unrelated GO terms (Figure S4). Upon closer inspection of module 5, in combination with the expression profile in EAC3 (Table S3) and literature, we identified several potential novel regulators of epicardial EMT and differentiation including *CRIP1*. *CRIP1* (or *CRP1*) encodes cysteine-rich protein 1, a protein that contains two LIM domains with associated glycine-rich repeats (Henderson et al., 1999). During cardiac development in zebrafish, CRIP1 (*csrp1*) is associated with altered cellular migration and behavior through non-canonical Wnt signaling (Miyasaka et al., 2007), which is a regulator of epicardial EMT (von Gise et al., 2011). Additionally, CRIP1 functions as a co-factor of serum response factor (SRF), another inducer of EMT in the epicardium during development (Trembley et al., 2015). To identify genes that are regulated by CRIP1, we inferred a gene regulatory network (GRN) using GENIE3 (Huynh-Thu et al., 2010). We used *WT1* as a benchmark since this transcription factor and its downstream targets have been characterized extensively. Interestingly, within the list of genes with the highest weight, we found several that are implied in epicardial differentiation (*TCF21*, *PDGFRa*) as well as marker of epicardial epithelium (*UPK3B*) to be associated with CRIP1 (Table S5). The expression profiles of *CRIP1* and its putative downstream targets *UPK3B* and *TCF21* were assessed. The pseudotime trajectory of *CRIP1* indicated a similar expression profile to *UPK3B* (high in EAC3, low in EAC2, 1, and 4). This profile is opposite to *TCF21,* which is highly expressed in the mesenchymal clusters (Figure 4E). BNC1 is known as a master regulator of epicardial phenotypes (Gambardella et al., 2019), and its expression was observed in the same module 5 as *CRIP1*. Indeed, while *BNC1* expression was low, it displayed a similar profile to *CRIP1*, with the largest change in EAC3 (Figure 4E). Moreover, *SNAI2,* a transcription factor involved in epicardial EMT, also has low expression at the start of the trajectory and has the largest change in expression in EAC3 and 2, after which it remains high in the mesenchymal clusters. These data could suggest a potential role for CRIP1 in the epicardium during development.

CRIP1 in epicardial EMT and developmental processes

Based on our sequencing and immunohistochemistry data, CRIP1 represents a marker of the epicardial epithelium. Considering its potential targets as a regulator based on GRN (Table S5), we sought to identify the function of CRIP1 in epicardial EMT and other key developmental processes. We established the presence of CRIP1 protein in the epicardium at 13 weeks of development (Figure 3B) and expanded this time window from 8 to 17 weeks of gestational age and in the adult. CRIP1 was epicardially expressed throughout cardiac development as well as in adult

Figure 4. Pseudotime analysis indicates differentiation from epithelial to mesenchymal populations

(A) Differentiation trajectories superimposed on tSNE map from Figure 2. Color of nodes: entropy. Color of links: –log10 p value. Width of links represents number of cells on the intercluster link.

(B) Stem cell score of clusters as calculated by ((number of links × entropy) – minimum median entropy across all clusters).

(C) Self-organizing map (SOM) of gene expression profiles (log2) on the differentiation trajectory from A. X axis shows cluster numbers. Module 5 is highlighted by dashed outline.

(D) GO term enrichment for genes in module 5 of the SOM in (C).

(E) Expression profiles of *CRIP1*, *UPK3B*, *BNC1, TCF21,* and *SNAI2* along the differentiation trajectory from (A). Expression is displayed as untransformed values. See also Figure S4.

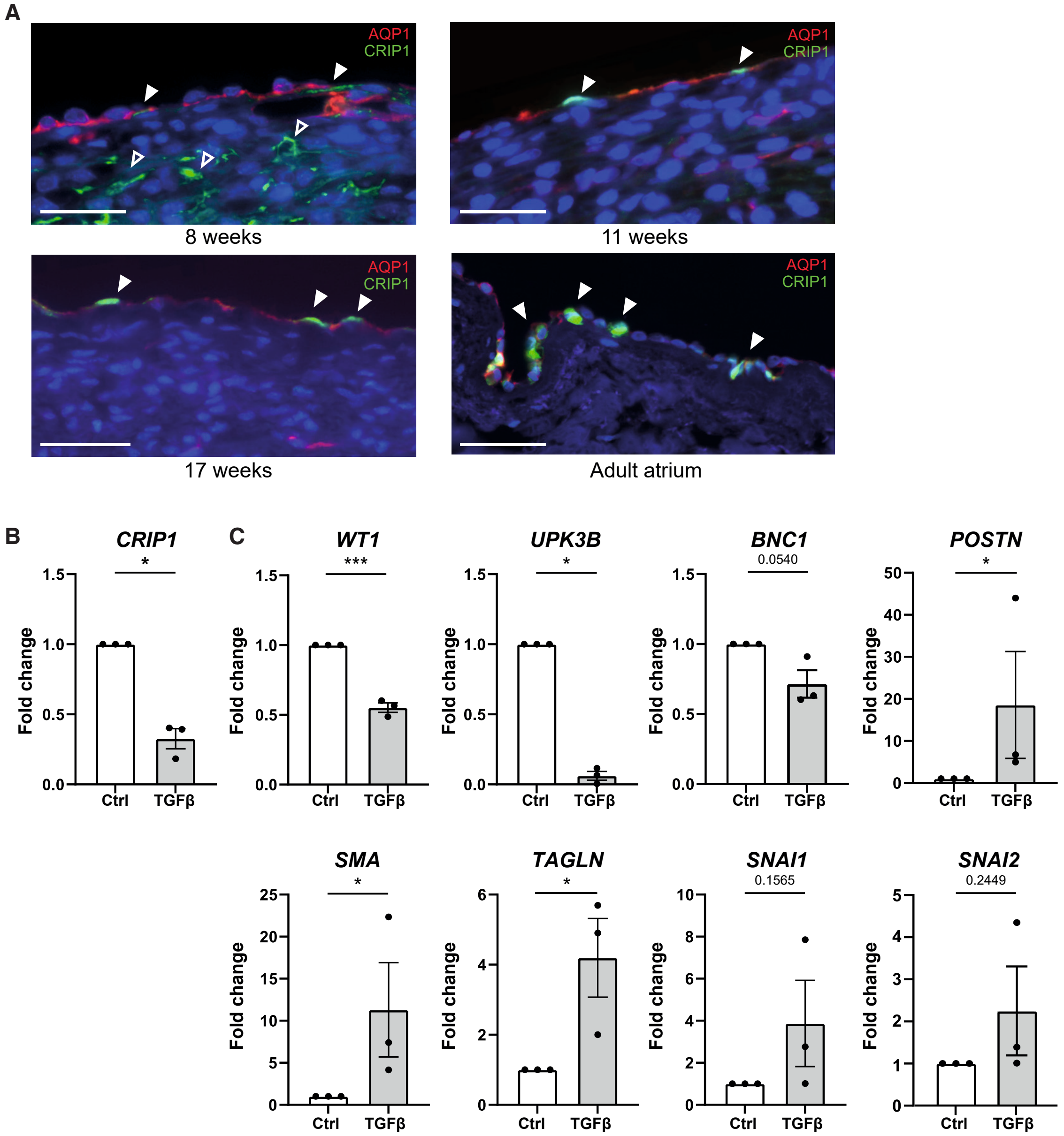

Figure 5. CRIP1 is a marker of human fetal epicardium and is downregulated in primary human cultured epicardial cells undergoing EMT

(A) Immunohistochemistry of CRIP1 during various stages of development and in adult atrial tissue. Solid arrowheads: epithelial expression. Open arrowheads: mesenchymal expression. Counterstain is DAPI. Scale bar: 25 μm.

(B) qPCR of various epicardial and mesenchymal markers after 5 days in control conditions or after the addition of TGFβ to induce EMT. Data are presented as mean ± SEM of three independent samples. *$p < 0.05$, ***$p < 0.001$; Student's t test.

atrial epicardium (Figure 5A). As anticipated, we also observed CRIP1 expression in the SMCs of coronary arteries (Figure S3B), as was previously shown in the adult murine heart (Henderson et al., 1999). To establish the role of CRIP1 in epicardial cells, a human epicardial cell culture model was used (Moerkamp et al., 2016). *In vitro,* primary epicardial cells retain their epithelial phenotype and undergo EMT after stimulation with, e.g., TGFβ, allowing controlled analysis of cellular processes. Using this model, we confirmed that *CRIP1* has a high expression in an epithelial state (control) and a reduced expression 5 days after TGFβ-induced EMT (Figure 5B). To confirm that our culture model followed general expression dynamics of the epicardium, the expression levels of *WT1,*

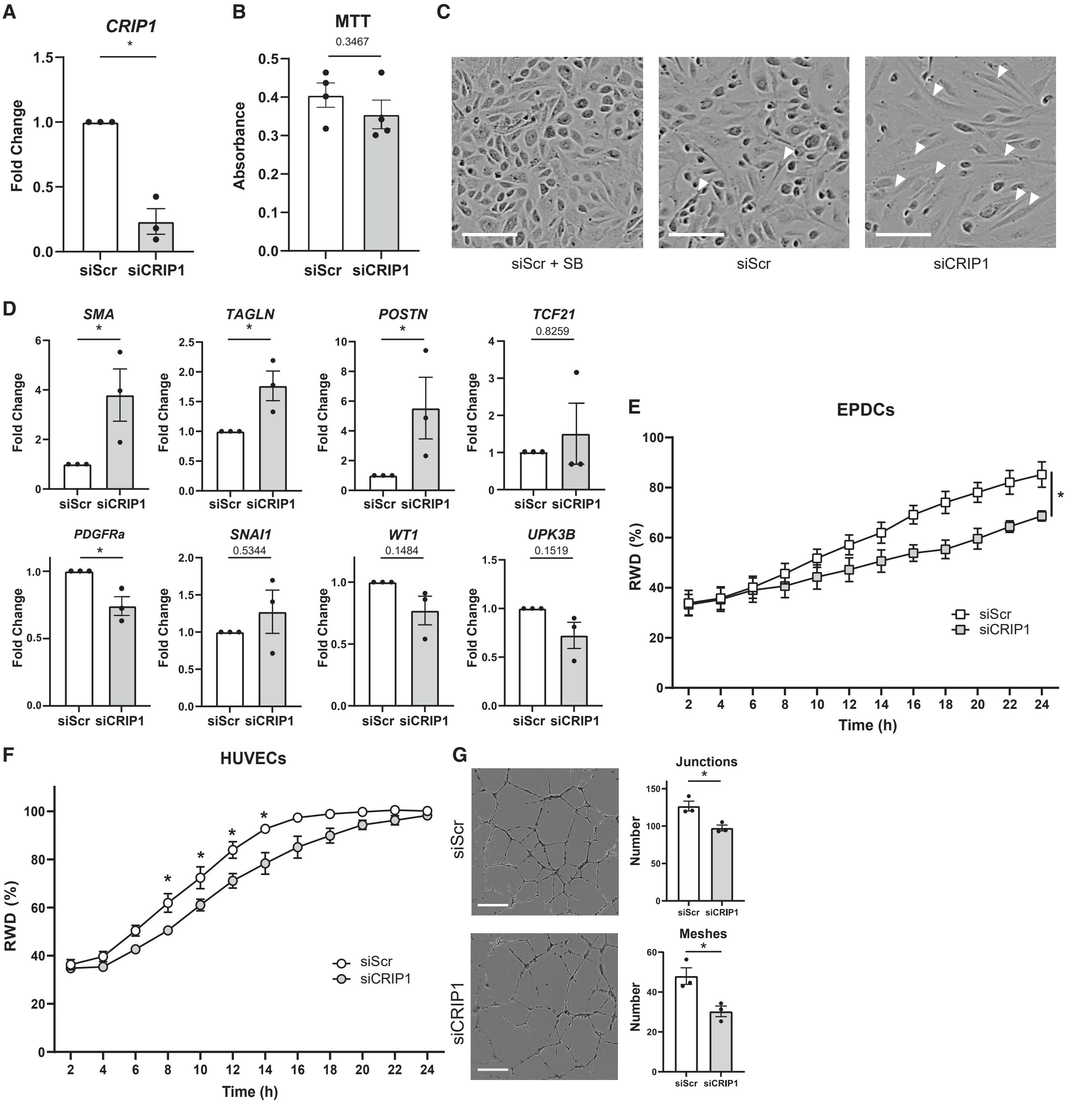

Figure 6. siRNA-induced knockdown of CRIP1 induces EMT in an epicardial cell culture model

(A) Validation of knockdown using qPCR. Data are presented as mean ± SEM of three independent samples. *$p < 0.05$; Student's t test.

(B) MTT assay to assess proliferation in siScr and siCRIP1 treated cells. Data are presented as mean ± SEM of four independent samples.

(C) Representative images of primary epicardial cells after 48 h cultured with scrambled siRNA (siScr) and SB to maintain an epithelial phenotype, siSCR alone, or siCRIP1. Arrowheads indicate examples of mesenchymal cells. Scale bar: 50 μm.

(D) qPCR for mesenchymal markers (*SMA, TAGLN, POSTN, TCF21, PDGFRa*), EMT-related transcription factor *SNAI1*, and epithelium (*UPK3B, WT1*). Data are presented as mean ± SEM of three independent samples. *$p < 0.05$; Student's t test.

(legend continued on next page)

UPK3B, and mesenchymal markers (*TAGLN, SMA, POSTN)* were assessed. Indeed, it showed that epicardial markers are downregulated, and mesenchymal genes were strongly upregulated 5 days after TGFβ induced EMT in our *in vitro* epicardial cell model (Figure 5C). The early EMT-related transcription factor *SNAI1* is likely no longer upregulated at this stage.

Since in our dataset (1) all epithelial cells express *CRIP1,* and (2) *CRIP1* is reduced once mesenchymal markers are expressed as well as upon the induction of EMT through TGFβ, we hypothesized that CRIP1 is required for the maintenance of an epithelial phenotype in human cardiac development.

To assess the role of CRIP1 in epicardial EMT, we performed siRNA-mediated knockdown (KD) of *CRIP1* in cultured primary epicardial cell isolations. *CRIP1* KD was validated by qPCR (Figure 6A), and KD did not affect proliferation of epicardial cells (Figure 6B). Interestingly, removal of *CRIP1* was sufficient to induce EMT already after 2 days shown by the appearance of spindle-shaped cells (Figure 6C). The change in cell morphology coincided with the increased expression of mesenchymal markers *SMA*, *TAGLN*, and *POSTN* (Figure 6D). The expression of *TCF21*, a marker of mesenchymal cells, had a limited increase in expression. Interestingly, the fibroblast-related marker *PDGFRa* was reduced upon treatment with siCRIP1, which may suggest that CRIP1 KD drives cells more toward a smooth muscle cell phenotype. Although the expression of EMT-related transcription factor *SNAI1* increased, this did not reach significance. Epithelial markers *WT1* and *UPK3B* were lowered upon KD but not yet significantly (Figure 6D). Since CRIP1 was identified as a regulator of TCF21, PDGFRa, and UPK3B (Table S5), we investigated whether a correlation existed between *CRIP1* expression and its targets identified in the GRN in the siCRIP1 cells. There was a weak correlation between *CRIP1* and *PDGFRa* (R^2 0.6922), but we found a strong negative correlation between *CRIP1* and *TCF21* (R^2 0.9933) and a positive correlation with *UPK3B* (R^2 0.9791), suggesting that *CRIP1* levels at least indirectly affect *TCF21* and *UPK3B* expression (Figure S5A).

Migration is a crucial process in epicardial development; therefore, we investigated whether CRIP1 was also involved in migration using a wound healing assay. *CRIP1* KD reduced the migration rate after 24 h (87.6 ± 2.9 vs. 72.1 ± 3.5) (Figures 6E and S5B).

In our total cell scRNA seq dataset, *CRIP1* was expressed in a subset of ECs (Figure S3B). To establish whether CRIP1 has a function in ECs, CRIP1 KD was performed in human umbilical vein endothelial cells (HUVECs). Similar to epicardial cells, KD of CRIP1 in HUVECs resulted in reduced migration (Figures 6F and S5C). Additionally, in a tube formation assay, CRIP1 influenced angiogenic properties in HUVECs; siCRIP1 reduced the number of junctions and meshes (Figure 6G). These data suggest that CRIP1 plays a role as regulator of multiple cardiac developmental processes, such as angiogenesis, but also in epicardial EMT and migration.

DISCUSSION

The epicardium plays a crucial role in heart development and may contribute to cardiac repair after injury. Detailed information on the human developing epicardium and its derivatives is limited. Here, we have characterized the human fetal epicardium on a single-cell level in an epicardial cell-enriched dataset. Using these data, we have (1) identified novel markers of the epicardium in epithelial and mesenchymal state and (2) investigated EMT resulting in a role for CRIP1 as a regulator of epicardial EMT.

Previous studies have relied on the analysis of the epicardium as a part of the single-cell data obtained from whole hearts. In many cases, the epicardium is represented by a very limited number of cells, and it is often identified merely on a few (known) markers. Others have isolated the epicardium and its derivatives by FACS based on established epicardial markers (e.g., WT1). However, neglecting cells that are negative for this marker may introduce a bias into the derived cell population. Here we show that specifically removing and processing the human fetal epicardial layers yields a strongly enriched cell population containing both epithelial-like epicardial cells and its derivatives (71.1%) without the limitations of using established markers.

Our dataset reveals that the established epicardial genes *WT1, TBX18*, and *TCF21* do not identify all epicardial associated cells on the mRNA level. In mouse embryos staged embryonic day 11.5 (E11.5), a great degree of overlap between *Wt1, Tbx18,* and *Tcf21* expression was observed, but these epicardial cells lost their marker signature as development progressed (Lupu et al., 2020). Since we

(E) Wound healing assay in primary epicardial culture after knockdown of CRIP1. RWD: relative wound density, n = 3 independent samples, *p < 0.05 at 24 h. Student's t test.
(F) Wound healing assay in HUVECs after knockdown of CRIP1, n = 3 independent samples. *p < 0.05; Student's t test.
(G) Images of tube formation by HUVECs after knockdown of CRIP1. Right: number of junctions and meshes in HUVECs after 24 h. Scale bar: 400 μm. Data are presented as mean ± SEM of three independent samples. *p < 0.05; Student's t test. See also Figure S5.

have investigated a relatively short developmental time span, there is a possibility that these genes have a higher degree of overlap in the human heart at earlier developmental stages. We have previously established that fetal epicardial cells from gestational age 14–15 weeks are very active and undergo spontaneous EMT (Moerkamp et al., 2016). Since the focus of this study was to investigate processes such as EMT and epicardial activity, this prompted us to focus on this developmental stage.

Over the last few years, heterogeneity within the epicardium has been studied with the aim to identify cells with specific abilities in a developmental and regenerative context. In our dataset, we found two very similar populations of epithelial cells, arguing against heterogeneity in the epicardium, which is in line with recent findings in mice (Lupu et al., 2020). Although in the mesenchymal cells there was persistent subclustering, we were unable to identify distinguishing markers for individual mesenchymal subpopulations. Moreover, GO enrichment analysis revealed no functional difference between these populations. This led us to conclude that these subpopulations may reflect the differentiation status rather than a different intrinsic functional capacity. The heterogeneity previously described by others could be explained by ongoing EMT or differentiation (Streef and Smits, 2021). Indeed, our most progenitor-like cluster (EAC3) already displays lower expression of the epithelium-related gene *UPK3B* and an increase in *TCF21* mRNA, suggesting that EMT is already initiated in this cluster. Our dataset revealed novel and specific markers of the human fetal epithelial epicardium and the mesenchymal population. For mesenchymal cells, we report for the first time the expression of NRK. *Nrk* has been reported to regulate expression of chemokines and matrix metalloproteinases in adult mouse SMCs (Lu et al., 2020). In skeletal muscle cells, NRK organizes cytoskeletal actin through actin polymerization (Nakano et al., 2003), which could be relevant in the differentiation of EPDCs since it leads to SRF-dependent transcription (Olson and Nordheim, 2010). In our dataset, NRK is very specific for mesenchymal cell populations. Interestingly, when analyzing the spatial distribution on a protein level, we observed NRK present within the subepicardium and in the outer epicardial layer. Since epicardial cells in the fetal heart are prone to undergo spontaneous EMT (Moerkamp et al., 2016), they may reside in the epithelial layer at an intermediate stage, which was reported previously (Mantri et al., 2021). We further confirmed this by identifying NRK and TCF21 in the outer epicardium, which may clarify some of the reported heterogeneity. The spontaneous tendency to undergo EMT may also explain why we find a relatively low number of fully epithelial cells (EAC3 and 8) in our dataset, as was also observed by Knight-Schrijver et al. (2022).

The dataset revealed CRIP1 as a novel marker present within the epithelial epicardium at week 14–15 of development. Protein expression within the epicardial layer was confirmed by immunohistochemistry throughout development and in the adult epicardium.

We continued to analyze CRIP1 based on its expression pattern and its GRN that included genes involved in epicardium (*UPK3B*) and epicardial differentiation (*TCF21*). When investigating the pseudotime trajectories, CRIP1 was suggested to be a potential regulator of epicardial EMT. In cell culture, we found that KD of *CRIP1* rapidly induced EMT, and it inhibited epicardial migration. The continuous expression of CRIP1 in the epicardium and its role in preventing EMT was initially surprising since CRIP1 has been proposed to be an inducer of EMT in cancer (He et al., 2017; Li et al., 2018). In idiopathic pulmonary fibrosis, CRIP1 was induced by TGFβ and highly expressed in fibroblasts (i.e., mesenchymal cells) (Järvinen et al., 2012). However, in agreement with our observations, a high CRIP1 expression in human breast cancer was associated with a better prognosis, and KD of CRIP1 increased the invasive potential of breast cancer cells *in vitro* (Ludyga et al., 2013), suggesting cell-type-specific functions of CRIP1. CRIP1 has been described as a co-factor of SRF, a known regulator of epicardial EMT (Trembley et al., 2015). SRF is a transcription factor with many different targets, including mitogen-responsive and muscle-specific genes. Both CRIP1 and the closely related CRIP2 have been described as SMC differentiation co-factors through interaction with SRF (Chang et al., 2003; Henderson et al., 1999). SRF activity is context dependent and is orchestrated through the binding of tissue-specific regulatory co-factors (Posern and Treisman, 2006), and as such, the function of CRIP1 could be different depending on the cellular context. Our dataset revealed that, besides the expression within the epicardium, *CRIP1* expression was also observed in other cells including ECs. KD of CRIP1 in HUVECs affected migration and angiogenic properties, indicating that CRIP1 has a function in several cell types that are crucial for cardiac development. The exact mechanism for CRIP1 in epicardial cells remains to be investigated, but based on tissue expression and the rapid induction of EMT upon CRIP1 removal, we anticipate it functions as an essential co-factor in maintaining epithelial homeostasis. Overall, knowledge of the human fetal epicardium is sparse due to the limited availability of tissue and the underrepresentation of the epicardium in most cardiac datasets. We provide extensive data of epicardial cells and their derivatives that allow a detailed investigation of the human fetal epicardium and its differentiation pathways.

EXPERIMENTAL PROCEDURES

Resource availability

Corresponding author

Further information and requests for resources and reagents should be directed to and will be fulfilled by the corresponding author, A.M.S. (a.m.smits@lumc.nl).

Materials availability

This study did not generate new unique reagents. Details about methods, reagents, cells, and scRNA-seq analyses can be found in the methods section and in the supplemental experimental procedures.

Data and code availability

Human fetal epicardium data have been deposited in the GEO data repository. The accession number for the human fetal epicardium sequencing data reported in this paper is GEO database: GSE213669. All transcriptomic analyses were performed using standard protocols with previously described R packages.

Collection of human cardiac tissue

Human adult heart auricles were collected anonymously as surgical waste from patients undergoing cardiac surgery under general informed consent. Human fetal cardiac tissue was anonymously collected with informed consent from elective abortion material of fetuses (gestational age 8 and 20 weeks). This research was carried out according to the official guidelines of the Leiden University Medical Center and approved by the local Medical Ethics Committee (No. P08.087). This research conforms to the Declaration of Helsinki.

Isolation of human epicardial cells

Fetal epicardial layers were isolated by separating the epicardium from the underlying myocardium of four human hearts aged 14–15 weeks post-gestation as previously described (Dronkers et al., 2018). After processing tissue into a single-cell suspension, cells were stained with 100 nM calcein AM (65–0853, Thermo Fisher).

Sorting and single-cell RNA library prep

Single-cell RNA library prep and plate preparation was performed by Single Cell Discoveries. Briefly, single viable cells were sorted into 384-well plates (one or two plates per sample, seven in total) filled with 50 nL lysis buffer containing CELseq2-primers, spike-ins, and dinucleotide triphosphates and immediately frozen at −80°C. cDNA was constructed using the SORT-seq protocol (Muraro et al., 2016). Detailed methods regarding sequencing and analysis with RaceID3, StemID2, and GENIE3 can be found in the supplemental experimental procedures.

Immunofluorescence staining of human heart tissue

Immunohistochemical staining was performed on formalin-fixed paraffin embedded human fetal and adult atrial cardiac tissue. 6-μm sections were incubated overnight at 4°C with primary antibodies: WT1 (ab89901, Abcam), AQP1 (sc-25287, Santa Cruz), CRIP1 (PA5-24643, Thermo Fisher), TCF21 (HPA013189, Sigma-Aldrich), NRK (PA-53566, Thermo Fisher), THBS4 (AF2390, R&D Systems), and SULF1 (ab32763, Abcam). TCF21 signal was amplified using TSA (NEL700A001KT, PerkinElmer). Appropriate secondary antibodies were used, and nuclei were stained with DAPI.

Isolation of mRNA and qPCR

mRNA was isolated using ReliaPrep RNA Miniprep Systems (Promega), followed by cDNA synthesis using the RevertAid H Minus First Strand cDNA Synthesis Kit (Thermo Fisher Scientific). qPCR was performed in a 384-well format using SYBR Green (Promega). Expression levels were normalized for two reference genes (HPRT1 and TBP); details and primer sequences are found in the supplemental experimental procedures.

Cell culture experiments

Primary epicardial cells were isolated and cultured as described previously (Moerkamp et al., 2016)). In experiments aimed at inducing EMT, cells were stimulated with 1 ng/mL transforming growth factor β 3 (TGFβ3, provided by Prof. P. ten Dijke). HUVECs were cultured in EGM2 (CC-3162, Lonza) supplemented with 2% FCS. siRNA experiments were performed using ON-TARGETplus SMARTPool siRNAs for CRIP1 (L-016212-00-0005, Dharmacon) or scrambled control (siScr, D-001810-10-05, Dharmacon) at 25 nM. For migration assays, cells were seeded at 20,000 cells per well. Detailed methods regarding cell culture and related experiments can be found in the supplemental experimental procedures.

Statistical analysis

Data are represented as mean ± SEM of at least three independent samples, and unpaired t test was used for statistical analysis unless otherwise stated. Statistical analysis was performed using GraphPad Prism 9.0.1.

SUPPLEMENTAL INFORMATION

Supplemental information can be found online at https://doi.org/10.1016/j.stemcr.2023.06.002.

AUTHOR CONTRIBUTIONS

Conceptualization, A.M.S.; methodology, T.S. and A.M.S.; investigation, T.S., E.J.G., and T.v.H.; software, T.S.; resources, J.H.; writing – original draft, T.S. and A.M.S.; writing – review & editing, A.M.S. and M.J.G.; supervision, A.M.S. and M.J.G.; funding acquisition, A.M.S.

ACKNOWLEDGMENTS

This work was supported by the Dutch Heart Foundation (Senior Dekker grant 2017T059 to A.M.S.).

CONFLICT OF INTERESTS

The authors declare no competing interests.

Received: November 15, 2022
Revised: June 2, 2023
Accepted: June 5, 2023
Published: June 29, 2023

REFERENCES

Acharya, A., Baek, S.T., Huang, G., Eskiocak, B., Goetsch, S., Sung, C.Y., Banfi, S., Sauer, M.F., Olsen, G.S., Duffield, J.S., et al. (2012). The bHLH transcription factor Tcf21 is required for lineage-specific EMT of cardiac fibroblast progenitors. Development *139*, 2139–2149. https://doi.org/10.1242/dev.079970.

Balmer, G.M., Bollini, S., Dubé, K.N., Martinez-Barbera, J.P., Williams, O., and Riley, P.R. (2014). Dynamic haematopoietic cell contribution to the developing and adult epicardium. Nat. Commun. *5*, 4054. https://doi.org/10.1038/ncomms5054.

Bochmann, L., Sarathchandra, P., Mori, F., Lara-Pezzi, E., Lazzaro, D., and Rosenthal, N. (2010). Revealing new mouse epicardial cell markers through transcriptomics. PLoS One *5*, e11429. https://doi.org/10.1371/journal.pone.0011429.

Braitsch, C.M., Combs, M.D., Quaggin, S.E., and Yutzey, K.E. (2012). Pod1/Tcf21 is regulated by retinoic acid signaling and inhibits differentiation of epicardium-derived cells into smooth muscle in the developing heart. Dev. Biol. *368*, 345–357. https://doi.org/10.1016/j.ydbio.2012.06.002.

Cai, C.-L., Martin, J.C., Sun, Y., Cui, L., Wang, L., Ouyang, K., Yang, L., Bu, L., Liang, X., Zhang, X., et al. (2008). A myocardial lineage derives from Tbx18 epicardial cells. Nature *454*, 104–108. https://doi.org/10.1038/nature06969.

Carmona, R., Barrena, S., López Gambero, A.J., Rojas, A., Muñoz-Chápuli, R., and Muñoz-Chápuli, R. (2020). Epicardial cell lineages and the origin of the coronary endothelium. FASEB J. *34*, 5223–5239. https://doi.org/10.1096/fj.201902249RR.

Chang, D.F., Belaguli, N.S., Iyer, D., Roberts, W.B., Wu, S.P., Dong, X.R., Marx, J.G., Moore, M.S., Beckerle, M.C., Majesky, M.W., et al. (2003). Cysteine-rich LIM-only proteins CRP1 and CRP2 are potent smooth muscle differentiation cofactors. Dev. Cell *4*, 107–118. https://doi.org/10.1016/S1534-5807(02)00396-9.

Dronkers, E., Moerkamp, A.T., van Herwaarden, T., Goumans, M.J., and Smits, A.M. (2018). The isolation and culture of primary epicardial cells derived from human adult and fetal heart specimens. J. Vis. Exp. *2018*, e57370. https://doi.org/10.3791/57370.

Duan, J., Gherghe, C., Liu, D., Hamlett, E., Srikantha, L., Rodgers, L., Regan, J.N., Rojas, M., Willis, M., Leask, A., et al. (2011). Wnt1/βcatenin injury response activates the epicardium and cardiac fibroblasts to promote cardiac repair. EMBO J. *31*, 429–442. https://doi.org/10.1038/emboj.2011.418.

Gambardella, L., McManus, S.A., Moignard, V., Sebukhan, D., Delaune, A., Andrews, S., Bernard, W.G., Morrison, M.A., Riley, P.R., Göttgens, B., et al. (2019). BNC1 regulates cell heterogeneity in human pluripotent stem cell-derived epicardium. Development *146*, dev174441. https://doi.org/10.1242/dev.174441.

von Gise, A., Zhou, B., Honor, L.B., Ma, Q., Petryk, A., and Pu, W.T. (2011). WT1 regulates epicardial epithelial to mesenchymal transition through beta-catenin and retinoic acid signaling pathways. Dev. Biol. *356*, 421–431. https://doi.org/10.1016/j.ydbio.2011.05.668.

Gittenberger-de Groot, A.C., Vrancken Peeters, M.P., Mentink, M.M., Gourdie, R.G., and Poelmann, R.E. (1998). Epicardium-derived cells contribute a novel population to the myocardial wall and the atrioventricular cushions. Circ. Res. *82*, 1043–1052. https://doi.org/10.1161/01.RES.82.10.1043.

Gittenberger-de Groot, A.C., Vrancken Peeters, M.P., Bergwerff, M., Mentink, M.M., and Poelmann, R.E. (2000). Epicardial outgrowth inhibition leads to compensatory mesothelial outflow tract collar and abnormal cardiac septation and coronary formation. Circ. Res. *87*, 969–971. https://doi.org/10.1161/01.RES.87.11.969.

Grieskamp, T., Rudat, C., Lüdtke, T.H.W., Norden, J., and Kispert, A. (2011). Notch signaling regulates smooth muscle differentiation of epicardium-derived cells. Circ. Res. *108*, 813–823. https://doi.org/10.1161/CIRCRESAHA.110.228809.

Guo, Y., and Pu, W.T. (2020). Cardiomyocyte maturation. Circ. Res. *126*, 1086–1106. https://doi.org/10.1161/CIRCRESAHA.119.315862.

He, G., Zou, L., Zhou, L., Gao, P., Qian, X., and Cui, J. (2017). Cysteine-Rich Intestinal Protein 1 Silencing Inhibits Migration and Invasion in Human Colorectal Cancer. Cell. Physiol. Biochem. *44*, 897–906. https://doi.org/10.1159/000485357.

Henderson, J.R., Macalma, T., Brown, D., Richardson, J.A., Olson, E.N., and Beckerle, M.C. (1999). The LIM protein, CRP1, is a smooth muscle marker. Dev. Dyn. *214*, 229–238. https://doi.org/10.1002/(SICI)1097-0177(199903)214:3.

Herman, J.S., Grün, D., and Grün, D. (2018). Fateid infers cell fate bias in multipotent progenitors from single-cell rnA-seq data. Nat. Methods *15*, 379–386. https://doi.org/10.1038/nMeth.4662.

Huynh-Thu, V.A., Irrthum, A., Wehenkel, L., and Geurts, P. (2010). Inferring regulatory networks from expression data using tree-based methods. PLoS One *5*, e12776. https://doi.org/10.1371/JOURNAL.PONE.0012776.

Järvinen, P.M., Myllärniemi, M., Liu, H., Moore, H.M., Leppäranta, O., Salmenkivi, K., Koli, K., Latonen, L., Band, A.M., and Laiho, M. (2012). Cysteine-rich protein 1 is regulated by transforming growth factor-β1 and expressed in lung fibrosis. J. Cell. Physiol. *227*, 2605–2612. https://doi.org/10.1002/jcp.23000.

Kikuchi, K., Gupta, V., Wang, J., Holdway, J.E., Wills, A.A., Fang, Y., and Poss, K.D. (2011). Tcf21+ epicardial cells adopt non-myocardial fates during zebrafish heart development and regeneration. Development *138*, 2895–2902. https://doi.org/10.1242/dev.067041.

Knight-Schrijver, V.R., Davaapil, H., Bayraktar, S., Ross, A.D.B., Kanemaru, K., Cranley, J., Dabrowska, M., Patel, M., Polanski, K., He, X., et al. (2022). A single-cell comparison of adult and fetal human epicardium defines the age-associated changes in epicardial activity. Nat. Cardiovasc. Res. *1*, 1215–1229. https://doi.org/10.1038/s44161-022-00183-w.

Li, H.G., Zhao, L.H., Zhang, Z.H., Liu, J.Z., Ren, K., Li, S.Y., and Su, Z.J. (2018). The Impact of Cysteine-Rich Intestinal Protein 1 (CRIP1) on Thyroid Carcinoma. Cell. Physiol. Biochem. *43*, 2037–2046. https://doi.org/10.1159/000484184.

Lu, Y.J., Jan, Y.J., Ko, B.S., Liang, S.M., Chen, L., Wu, C.C., Chin, C.H., Kuo, C.C., Yet, S.F., and Liou, J.Y. (2020). Expression of Nik-related kinase in smooth muscle cells attenuates vascular inflammation and intimal hyperplasia. Aging *12*, 7511–7533. https://doi.org/10.18632/AGING.103104.

Ludyga, N., Englert, S., Pflieger, K., Rauser, S., Braselmann, H., Walch, A., Auer, G., Höfler, H., and Aubele, M. (2013). The impact of Cysteine-Rich Intestinal Protein 1 (CRIP1) in human breast cancer. Mol. Cancer *12*, 28. https://doi.org/10.1186/1476-4598-12-28.

Lupu, I.E., Redpath, A.N., and Smart, N. (2020). Spatiotemporal analysis reveals overlap of key proepicardial markers in the developing murine heart. Stem Cell Rep. *14*, 770–787. https://doi.org/10.1016/j.stemcr.2020.04.002.

Mantri, M., Scuderi, G.J., Abedini-Nassab, R., Wang, M.F.Z., McKellar, D., Shi, H., Grodner, B., Butcher, J.T., and De Vlaminck, I. (2021). Spatiotemporal single-cell RNA sequencing of developing chicken hearts identifies interplay between cellular differentiation and morphogenesis. Nat. Commun. *12*, 1771. https://doi.org/10.1038/s41467-021-21892-z.

Masters, M., and Riley, P.R. (2014). The epicardium signals the way towards heart regeneration. Stem Cell Res. *13*, 683–692. https://doi.org/10.1016/j.scr.2014.04.007.

Miyasaka, K.Y., Kida, Y.S., Sato, T., Minami, M., and Ogura, T. (2007). Csrp1 regulates dynamic cell movements of the mesendoderm and cardiac mesoderm through interactions with Dishevelled and Diversin. Proc. Natl. Acad. Sci. USA *104*, 11274–11279. https://doi.org/10.1073/pnas.0702000104.

Moerkamp, A.T., Lodder, K., Van Herwaarden, T., Dronkers, E., Dingenouts, C.K.E., Tengström, F.C., Van Brakel, T.J., Goumans, M.J., and Smits, A.M. (2016). Human fetal and adult epicardial-derived cells: a novel model to study their activation. Stem Cell Res. Ther. *7*, 174. https://doi.org/10.1186/s13287-016-0434-9.

Moore, A.W., Schedl, A., McInnes, L., Doyle, M., Hecksher-Sorensen, J., and Hastie, N.D. (1998). YAC transgenic analysis reveals Wilms' tumour 1 gene activity in the proliferating coelomic epithelium, developing diaphragm and limb. Mech. Dev. *79*, 169–184. https://doi.org/10.1016/S0925-4773(98)00188-9.

Moore-Morris, T., Cattaneo, P., Puceat, M., and Evans, S.M. (2016). Origins of cardiac fibroblasts. J. Mol. Cell. Cardiol. *91*, 1–5. https://doi.org/10.1016/j.yjmcc.2015.12.031.

Muraro, M.J., Dharmadhikari, G., Grün, D., Groen, N., Dielen, T., Jansen, E., van Gurp, L., Engelse, M.A., Carlotti, F., de Koning, E.J.P., et al. (2016). A single-cell transcriptome atlas of the human pancreas. Cell Syst. *3*, 385–394.e3. https://doi.org/10.1016/J.CELS.2016.09.002.

Nakano, K., Kanai-Azuma, M., Kanai, Y., Moriyama, K., Yazaki, K., Hayashi, Y., and Kitamura, N. (2003). Cofilin phosphorylation and actin polymerization by NRK/NESK, a member of the germinal center kinase family. Exp. Cell Res. *287*, 219–227. https://doi.org/10.1016/S0014-4827(03)00136-8.

Niderla-Bielinska, J., Jankowska-Steifer, E., Flaht-Zabost, A., Gula, G., Czarnowska, E., and Ratajska, A. (2019). Proepicardium: current understanding of its structure, induction, and fate. Anat. Rec. *302*, 893–903. https://doi.org/10.1002/ar.24028.

Olson, E.N., and Nordheim, A. (2010). Linking actin dynamics and gene transcription to drive cellular motile functions. Nat. Rev. Mol. Cell Biol. *11*, 353–365. https://doi.org/10.1038/nrm2890.

Posern, G., and Treisman, R. (2006). Actin' together: serum response factor, its cofactors and the link to signal transduction. Trends Cell Biol. *16*, 588–596. https://doi.org/10.1016/j.tcb.2006.09.008.

Smart, N., Bollini, S., Dubé, K.N., Vieira, J.M., Zhou, B., Davidson, S., Yellon, D., Riegler, J., Price, A.N., Lythgoe, M.F., et al. (2011). De novo cardiomyocytes from within the activated adult heart after injury. Nature *474*, 640–644. https://doi.org/10.1038/nature10188.

Smith, C.L., Baek, S.T., Sung, C.Y., and Tallquist, M.D. (2011). Epicardial-derived cell epithelial-to-mesenchymal transition and fate specification require PDGF receptor signaling. Circ. Res. *108*, e15–e26. https://doi.org/10.1161/CIRCRESAHA.110.235531.

Streef, T.J., and Smits, A.M. (2021). Epicardial contribution to the developing and injured heart: exploring the cellular composition of the epicardium. Front. Cardiovasc. Med. *8*, 750243. https://doi.org/10.3389/fcvm.2021.750243.

Tian, X., Pu, W.T., and Zhou, B. (2015). Cellular origin and developmental program of coronary angiogenesis. Circ. Res. *116*, 515–530. https://doi.org/10.1161/CIRCRESAHA.116.305097.

Trembley, M.A., Velasquez, L.S., de Mesy Bentley, K.L., and Small, E.M. (2015). Myocardin-related transcription factors control the motility of epicardium-derived cells and the maturation of coronary vessels. Development *142*, 21–30. https://doi.org/10.1242/dev.116418.

Weinberger, M., Simões, F.C., Patient, R., Sauka-Spengler, T., and Riley, P.R. (2020). Functional heterogeneity within the developing zebrafish epicardium. Dev. Cell *52*, 574–590.e6. https://doi.org/10.1016/J.DEVCEL.2020.01.023.

Xia, Y., Duca, S., Perder, B., Dündar, F., Zumbo, P., Qiu, M., Yao, J., Cao, Y., Harrison, M.R.M., Zangi, L., et al. (2022). Activation of a transient progenitor state in the epicardium is required for zebrafish heart regeneration. Nat. Commun. *13*, 7704. https://doi.org/10.1038/s41467-022-35433-9.

Xu, C., Gao, X., Wei, Q., Nakahara, F., Zimmerman, S.E., Mar, J., and Frenette, P.S. (2018). Stem cell factor is selectively secreted by arterial endothelial cells in bone marrow. Nat. Commun. *9*, 2449. https://doi.org/10.1038/s41467-018-04726-3.

Zhou, B., Ma, Q., Rajagopal, S., Wu, S.M., Domian, I., Rivera-Feliciano, J., Jiang, D., von Gise, A., Ikeda, S., Chien, K.R., et al. (2008). Epicardial progenitors contribute to the cardiomyocyte lineage in the developing heart. Nature *454*, 109–113. https://doi.org/10.1038/nature07060.

Zhou, B., Honor, L.B., He, H., Ma, Q., Oh, J.H., Butterfield, C., Lin, R.Z., Melero-martin, J.M., Dolmatova, E., Duffy, H.S., et al. (2011). Adult mouse epicardium modulates myocardial injury by secreting paracrine factors. J. Clin. Invest. *121*, 1894–1904. https://doi.org/10.1172/JCI45529DS1.

Stem Cell Reports, Volume *18*

Supplemental Information

Single-cell analysis of human fetal epicardium reveals its cellular composition and identifies CRIP1 as a modulator of EMT

Thomas J. Streef, Esmee J. Groeneveld, Tessa van Herwaarden, Jesper Hjortnaes, Marie José Goumans, and Anke M. Smits

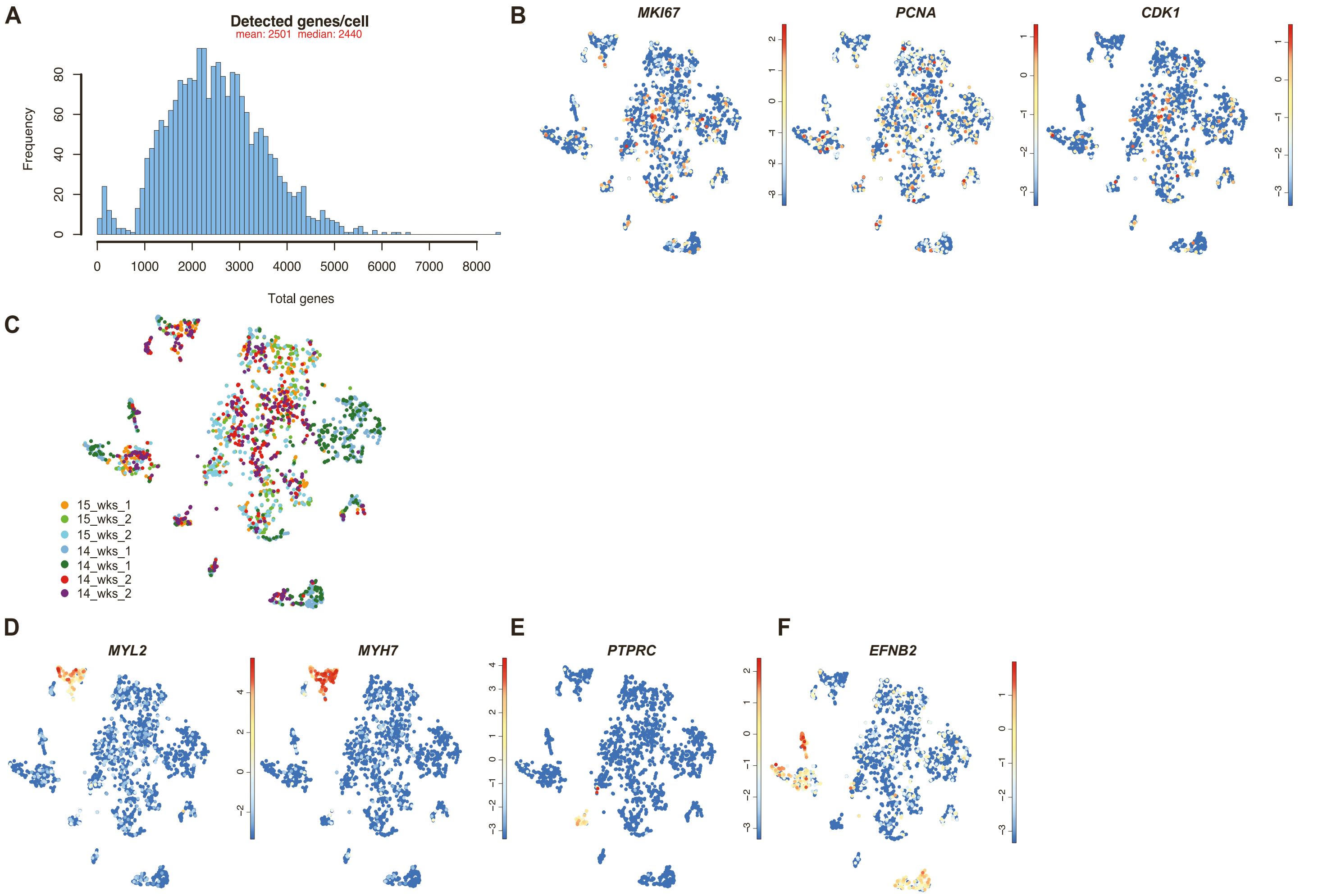

Figure S1. Analysis of clusters in scRNA seq data of isolated cells. Related to Figure 1
(A) Detected genes per cell over all samples. (B) tSNE map of expression of cell-cycle related genes. (C) Distribution of samples across clusters. (D) Expression of markers associated with mature cardiomyocytes. (E) Expression of CD45 (*PTPRC*). (F) Expression of *EFNB2.*

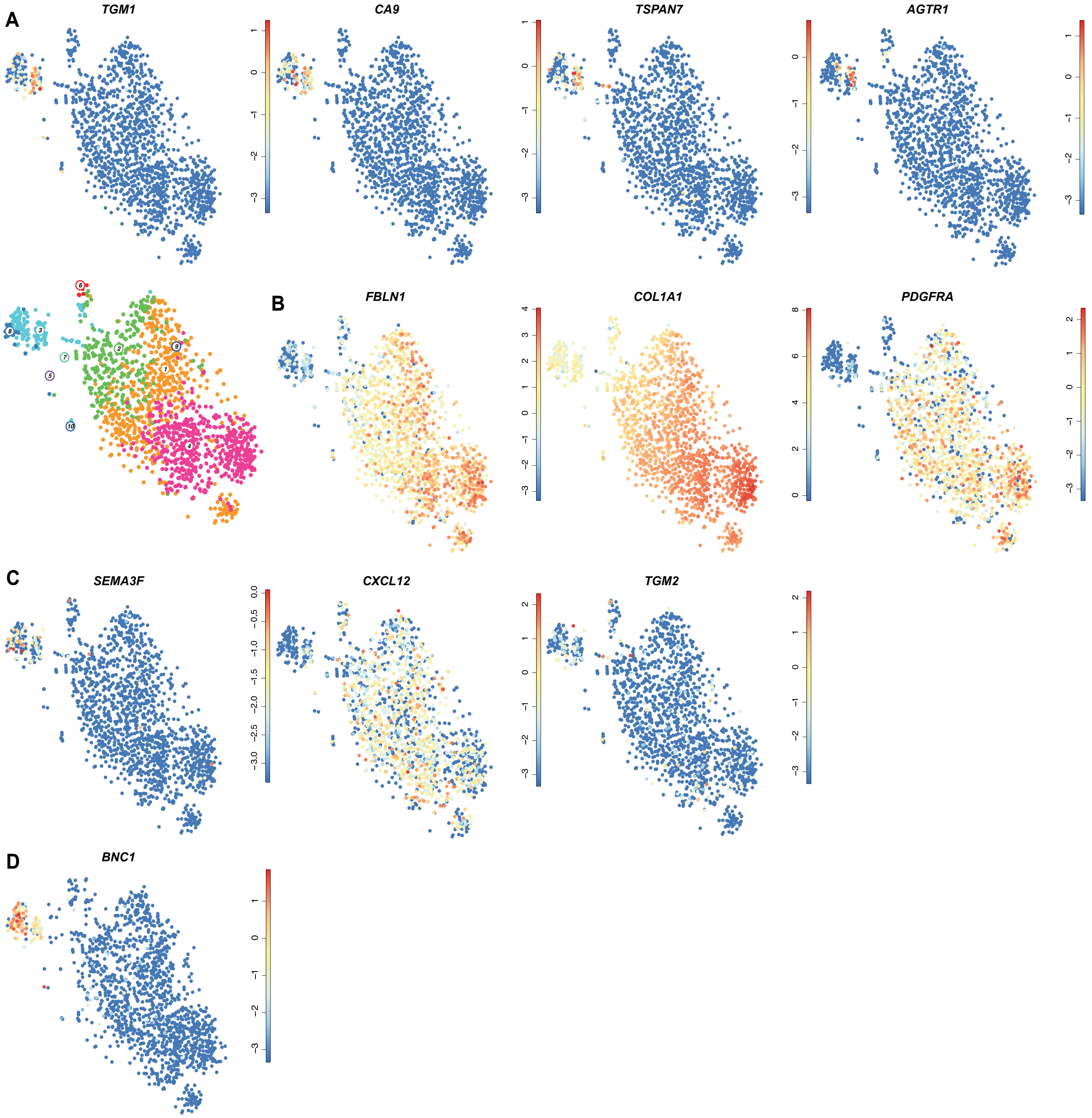

Figure S2. Expression of genes found in subclusters of EACs. Related to Figure 2.
(A) Expression of of markers found in EAC3: *TGM1, CA9, TSPAN7* and *AGTR1*. tSNE map of clustering serves as reference. (B) Expression of ECM-related genes and *PDGFRa* in EACs. (C) Expression of markers for subpopulations found in zebrafish *CXCL12*, *SEMA3F* and *TGM2*. (D) Expression of *BNC1*.

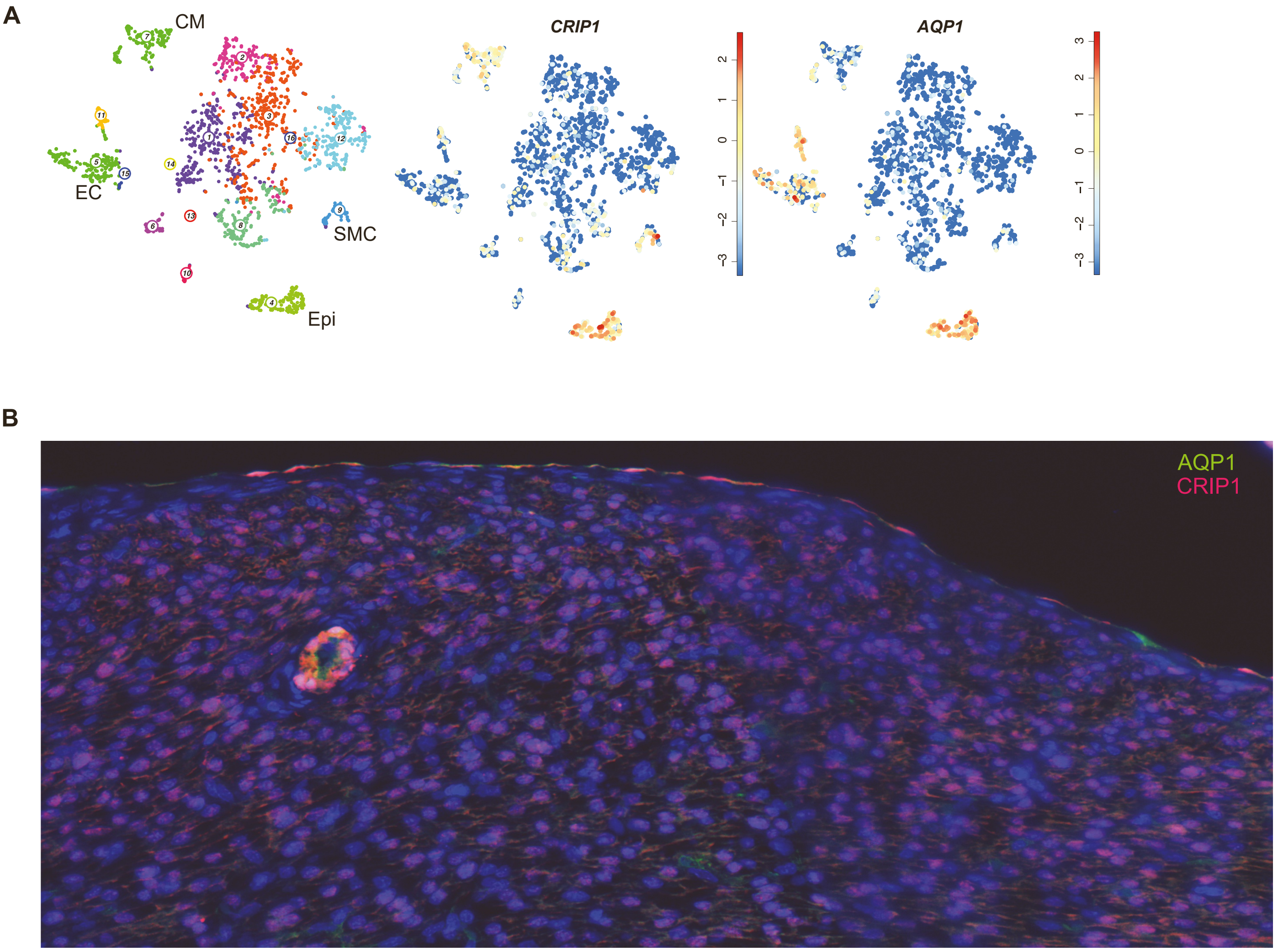

Figure S3. Expression on mRNA and protein level of AQP1 and CRIP1 in human fetal heart. Related to Fig 3.
(A) tSNE map of all clusters and expression of *CRIP1* and *AQP1*. tSNE map of all clusters serves as reference.
(B) Overview of immunohistochemistry of a section of human fetal heart tissue (13 weeks). Counterstain is DAPI.

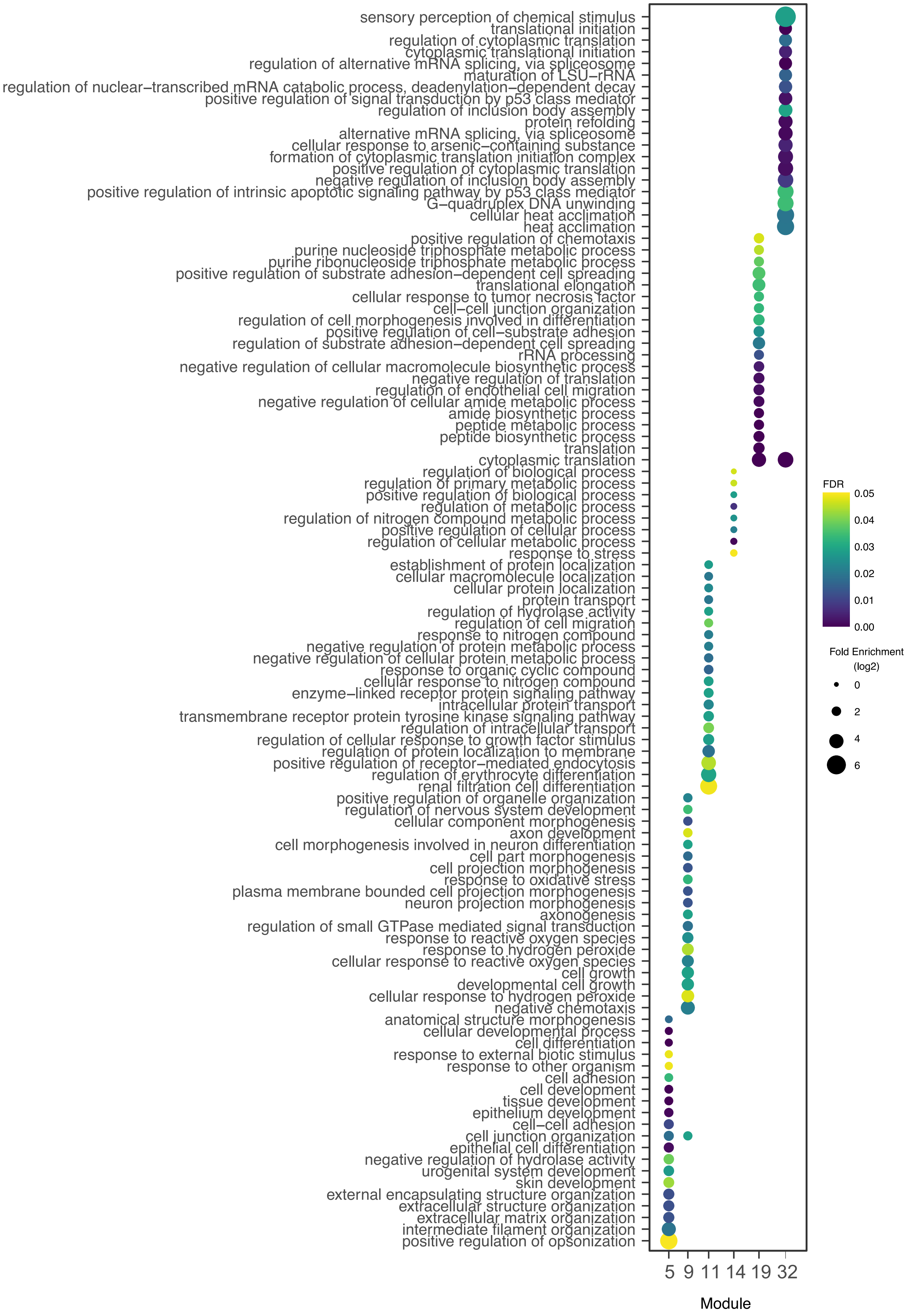

Figure S4. Gene ontology enrichment analysis of genes that have similar expression profile in the pseudotime trajectory. Related to Fig 4. GO term enrichment of genes found in gene expression modules with high expression in epithelial cells and low expression in mesenchymal cells. Color: false discovery rate (FDR), size: enrichment ratio (log2). Modules 1, 2, 3, 6, 7, 10, 12, 13, 15, 16 did not yield any significant GO term enrichment. Modules 4 and 8 only yielded 1 and 5 terms, respectively (data not shown). Top 20 terms with highest fold enrichment are displayed with FDR < 0.05.

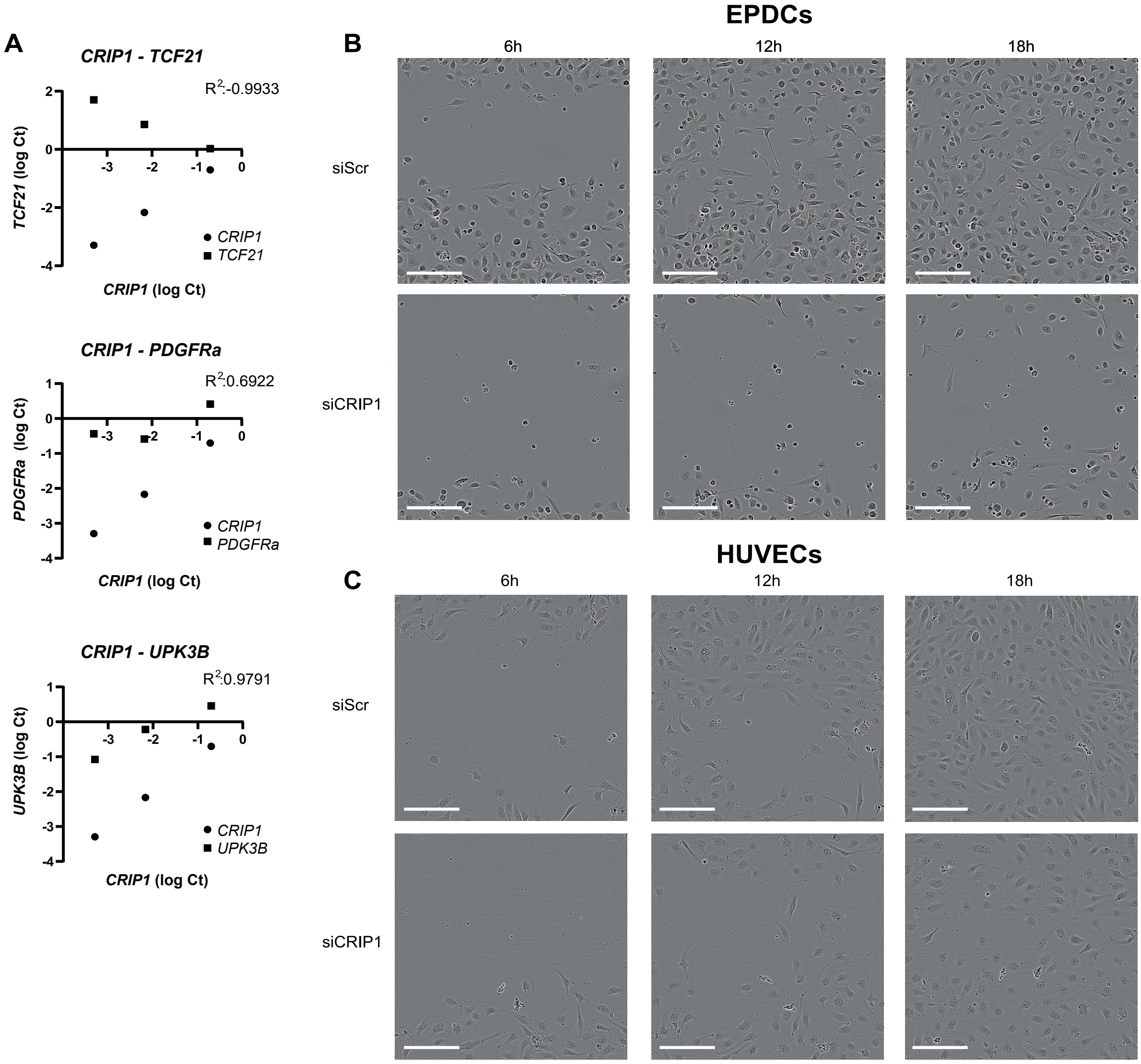

Figure S5. In vitro analysis of the effect of CRIP1 knockdown in EPDCs and HUVECs. Related to Fig 6.
(A)Correlation between *CRIP1* expression and *TCF21, PDGFRa, and UPK3B* in siCRIP1 cells.
(B) Phase contrast images of primary epicardial cells in wound healing assay 6h, 12h and 18h after scratch. Scale bars : 200 µm
(C) Phase contrast images of HUVECs in wound healing assay after 6h, 12h and 18h. Scale bars : 200 µm.

Supplemental experimental procedures

Collection of human cardiac tissue

Human adult heart auricles were collected anonymously as surgical waste from patients undergoing cardiac surgery under general informed consent. Human fetal cardiac tissue was anonymously collected with informed consent from elective abortion material of fetuses with a gestational age between 8 and 20 weeks. This research was carried out according to the official guidelines of the Leiden University Medical Center and approved by the local Medical Ethics Committee (number P08.087). This research conforms to the Declaration of Helsinki.

Isolation of human epicardial cells

Fetal epicardial layers were isolated by separating the epicardium from the underlying myocardium of 4 human hearts aged 14-15 weeks. To create a single cell suspension, the isolated tissue was processed into small pieces and digested during three rounds of Trypsin 0.25%/EDTA incubation (1:1; Serva and USH products) for a total of 30 minutes at 37 °C. The suspension was subsequently passed through a series of syringes of decreasing size (19G to 21G). The cell suspension was passed through a 70-µm cell strainer (BD Falcon), collected and stained for 20 minutes using calcein AM (65-0853, ThermoFisher,) at 100 nM in PBS at RT and subsequently kept on ice until sorting in 384-wells plates.

Sorting and single-cell RNA library prep

Single-cell RNA library prep and plate preparation was performed by Single Cell Discoveries. In short, using a Mosquito® HTS (TTP Labtech) 384 wells plates were filled with 50 nL lysis buffer containing CELseq2-primers, spike-ins and dinucleotide triphosphates and overlaid with mineral oil to prevent evaporation. Plates were stored at -80°C until sequencing. Viable cells were sorted one cell per well into these 384 wells plates using BD FACSAria III, fixed by lysis to preserve expression status, and immediately frozen at -80°C until further processing. cDNA was constructed using the SORT-seq protocol (Muraro et al., 2016).

Analysis of single-cell transcriptome data

RaceID3 was used according to authors instruction (Herman and Grün, 2018). Based on the distribution of the log10 total reads plotted against the frequency, we maintained a cutoff at minimally 1500 reads per cell before further analysis. Moreover, we discarded genes that did not express 3 transcripts in at least 3 cells. We initialized CGenes with *CDK1*, *PCNA*, *MKI67* and ccor = 0.4 to remove cell-cycle associated variability. RaceID3's internal batch correction was applied. To detect outliers, 'findoutliers' was initialized with probthr = 10^{-9}, outlg = 5, outminc = 3. For analysis of subset of epicardial cells, same parameters were applied. 'findoutliers' was run with same parameters, only probthr was set as 10^{-7}.

Gene ontology

Over-representation analysis of differentially expressed genes (fold change > 1.5) for each cluster (or all genes in cases of expression modules) were performed using the non-redundant Biological Process option of Gene Ontology (Ashburner et al., 2000; Carbon et al., 2021). Visualization was done using ggplot2.

Inference of differentiation trajectories

The StemID2 algorithm was used as published previously (Herman and Grün, 2018). In short, StemID2 is an algorithm for identification of differentiation trajectories by inferring links between cell clusters which are more populated by cells with intermediate single-cell transcriptomes than expected by chance. StemID calculates all pairwise cell-to-cell distances (1 – Pearson correlation) and uses this to cluster similar cells into clusters that correspond to the cell types present in the tissue. The StemID algorithm then calculates the number of links between clusters. StemID2 was initialized with cthr = 5, knn = 3 in *nmode.* To define stem cell populations, stem cell score was calculated by StemID2: stem cell score was calculated by multiplying the number of links with the delta-entropy ((number of links * entropy)- minimum median entropy across all clusters)).

Inference of co-expressed gene modules

To identify modules of co-expressed genes along a specific differentiation trajectory, FateID was used (Herman and Grün, 2018). In short, to identify co-expression of genes along a differentiation trajectory to a defined target state (as identified by StemID2), the expression levels in the pseudo-temporally

ordered cells are smoothed by local regression after z-score transformation. These pseudo-temporal gene expression profiles are topologically ordered through computation of a one-dimensional self-organizing map with 1000 nodes. Because of the large number of nodes relative to the number of clustered profiles, similar profiles are assigned to the same node. Only nodes with more than five assigned profiles are retained for visualization of co-expressed gene modules. Neighboring nodes with average profiles with a Pearson's correlation coefficient > 0.85 are merged with common gene expression modules. All functions were initialized with default values.

Inference of a gene regulatory network
GENIE3 computes the regulatory network for each gene independently. It calculates a random forest using genes as predictors. Then, it uses the random forests importance measure as gene association score to indicate a putative regulatory link. The weight of an interaction comes from the importance of an input gene in the predictor for a target gene's expression pattern. Aggregating these weighted interactions over all the genes yields the regulatory network. Genes with a median expression of 0 across all clusters were excluded.

Epicardial cell culture
Epicardial cells for cell culture were isolated as described above and cultured in a mixture of 1:1 Dulbecco's modified Eagle's medium (DMEM-glucose low; Invitrogen) and Medium 199 (M199; Invitrogen) supplemented with 10% heat-inactivated fetal calf serum (FCS; Gibco) and 100 U/ml penicillin/ streptomycin (Gibco). To maintain cells in an epithelial state, the ALK5-kinase inhibitor SB431542 (SB, 5–10 μM; Tocris Bioscience) was added. In experiments aimed at inducing EMT, cells were stimulated with 1 ng/ml transforming growth factor beta 3 (TGFβ3), without SB, for 5 days. TGFβ3 was kindly provided by Prof. P. ten Dijke.

HUVEC cell culture
Human umbilical vein endothelial cells (HUVECs) were cultured in EGM2 (CC-3162, Lonza) supplemented with 2% FCS and 100 U/ml penicillin/streptomycin until confluent and subsequently passaged.

Immunofluorescence staining of human heart tissue
Immunohistochemical staining was performed on formalin-fixed paraffin embedded human fetal and adult atrial cardiac tissue. Tissue was fixed overnight in 4% PFA and then processed for paraffin embedding and sectioned. 6 μm sections were deparaffinized and rehydrated by ethanol gradient and heat-induced antigen retrieval was performed in a pressure cooker for 20 minutes. Sections were washed in PBS/0.1% Tween20 and blocked with block buffer (PBS/2%BSA/0.1% Tween20) for 30 minutes. Sections were incubated with primary antibodies overnight at 4°C diluted in block buffer. Antibodies: WT1 (ab89901, Abcam), AQP1 (sc-25287, Santa Cruz), CRIP1 (PA5-24643, ThermoFisher), TCF21 (HPA013189, Sigma Aldrich), NRK (PA-53566, ThermoFisher), THBS4 (AF2390, R&D Systems), SULF1 (ab32763, Abcam). TCF21 signal was amplified using TSA (NEL700A001KT, PerkinElmer). Secondary antibodies were Donkey Anti-Mouse 555 (A-31570, Life Technologies) and Donkey Anti-Rabbit 647 (A-31572, Life Technologies). Nuclei were stained using DAPI (62248, ThermoFisher Scientific) and slides were mounted using Prolong Gold Antifade (P3630, Invitrogen)

Isolation of mRNA and qPCR
mRNA was isolated using ReliaPrep™ RNA Miniprep Systems (Promega). The mRNA concentration and purity were measured using NanoDrop 1000 Spectrophotometer (Thermo Fisher Scientific) followed by cDNA synthesis using the RevertAid H Minus First Strand cDNA Synthesis Kit (Thermo Fisher Scientific). qPCR was performed in a 384 wells format using SYBR Green (Promega) and run on a CFX384 Touch™ Real-Time PCR Detection System (Bio-Rad). Expression levels were normalized for two reference genes (*HPRT1* and *TBP*) which were designed and tested for robust expression in adult EPDCs and in epithelial and mesenchymal samples using geNorm (Vandesompele et al., 2002). Primers used are displayed below.

Target	Forward (5' - 3')	Reverse (5' - 3')
WT1	CAGCTTGAATGCATGACCTG	TATTCTGTATTGGGCTCCGC
TCF21	CAAGGCCTTCTCCAGACTCA	TCAGGTCACTCTCGGGTTTC
PDGFRa	GAGTGACCATCCTGCTGTGG	GTCTGCGAGCTGTGTCTGTT

POSTN	GACTCAAGATGATTCCCTTTTTACC	GCAACATTCATATAACACAGTCGTTT
SNAI1	CCAGTGCCTCGACCACTATG	CTGCTGGAAGGTAAACTCTGGA
SNAI2	CGGACCCACACATTACCTTGT	TTCTCCCCCGTGTGAGTTCTA
TAGLN	TTCAAGCAGATGGAGCAGGT	TTCAAGCAGATGGAGCAGGT
SMA	CCGGGAGAAAATGACTCAAA	GAAGGAATAGCCACGCTCAG
CRIP1	TCATGCCCAAGTGTCCCAAG	GGTTTGCCTTCGTGCTCAG
BNC1	CCACCGTCAGTGTGACCAAT	CAATCTCCACCTGGCTTGTT
TBP	TGGAAAAGTTGTATTAACAGGTGCT	GCAAGGGTACATGAGAGCCA
HPRT1	CTCATGGACTGATTATGGACAGGAC	GCAGGTCAGCAAAGAACTTATAGCC

siRNA experiments
Prior to adding siRNAs, SB431542 was removed to prevent interference in EMT. ON-TARGETplus SMARTPool siRNAs for CRIP1 (L-016212-00-0005, Dharmacon) or scrambled control (siScr, D-001810-10-05, Dharmacon) were transfected at 25 nM according to manufacturer's instruction without penicillin/streptomycin. After 24h incubation, medium was replaced by standard culture medium supplemented with FCS/penicillin/streptomycin and cells were stimulated for EMT experiments or replated at desired densities for migration experiments.

Migration experiments
Cells were seeded at 20.000 cells per well in a 96-wells plate (Corning) until confluent. Next, WoundMaker™ tool (Essen BioScience) was used to create reproducible scratches in all wells according to manufacturer's instructions. Cells were incubated for 24h in IncuCyte ZOOM™ and photos were taken every two hours. Analysis was performed using IncuCyte Scratch Wound Analysis software module. Statistical analysis for migration in HUVECs was performed with a paired student's t-test.

MTT
Proliferation after siRNA treatment was measured using MTT assay. After siRNA treatment for 24h, 7500 cells were seeded in a 96-wells plate for 24h. 3h prior to analysis, medium was refreshed with EPDC medium with 10% MTT (M5655, Sigma-Aldrich) solution. DMSO was added at the end of the 24h and absorbance was measured in Victor X3 (PerkinElmer) at 595 nm.

Tube formation
Prior to seeding HUVECs, 50 µl of Matrigel (Growth Factor Reduced, 354230, Corning) was incubated in 96-wells plates for 30 minutes. Cells were trypsinized after 24h of incubation with siRNAs (see siRNA interference), and 10.000 cells per well were seeded onto polymerized matrix in normal culture medium. Cells were incubated for 24h in IncuCyte ZOOM™ and photos were taken every two hours. Tube formation was stable at 24h, and images from this timepoint were analyzed with Image J 2.3.0 using Angiogenesis Analyzer (Carpentier et al., 2020).

References

Ashburner, M., Ball, C.A., Blake, J.A., Botstein, D., Butler, H., Cherry, J.M., Davis, A.P., Dolinski, K., Dwight, S.S., Eppig, J.T., et al. (2000). Gene Ontology: tool for the unification of biology. Nat. Genet. *25*, 25–29. https://doi.org/10.1038/75556

Carbon, S., Douglass, E., Good, B.M., Unni, D.R., Harris, N.L., Mungall, C.J., Basu, S., Chisholm, R.L., Dodson, R.J., Hartline, E., et al. (2021). The Gene Ontology resource: enriching a GOld mine. Nucleic Acids Res. *49*, D325–D334. https://doi.org/10.1093/NAR/GKAA1113.

Carpentier, G., Berndt, S., Ferratge, S., Rasband, W., Cuendet, M., Uzan, G., and Albanese, P. (2020). Angiogenesis Analyzer for ImageJ — A comparative morphometric analysis of "Endothelial Tube Formation Assay" and "Fibrin Bead Assay." Sci. Rep. *10*, 1–13. https://doi.org/10.1038/s41598-020-67289-8.

Herman, J.S., Sagar, and Grün, D. (2018). FateID infers cell fate bias in multipotent progenitors from single-cell RNA-seq data. Nat. Methods *15*, 379–386. https://doi.org/10.1038/nmeth.4662.

Muraro, M.J., Dharmadhikari, G., Grün, D., Groen, N., Dielen, T., Jansen, E., van Gurp, L., Engelse, M.A., Carlotti, F., de Koning, E.J.P., et al. (2016). A Single-Cell Transcriptome Atlas of the Human Pancreas. Cell Syst. *3*, 385-394.e3. https://doi.org/10.1016/J.CELS.2016.09.002.

Vandesompele, J., De Preter, K., Pattyn, F., Poppe, B., Van Roy, N., De Paepe, A., and Speleman, F. (2002). Accurate normalization of real-time quantitative RT-PCR data by geometric averaging of multiple internal control genes. Genome Biol. *3*, 1–12. https://doi.org/10.1186/GB-2002-3-7-RESEARCH0034/COMMENTS.
